# Computational analysis of Cyclin D1 gene SNPs and association with breast cancer

**DOI:** 10.1042/BSR20202269

**Published:** 2021-01-29

**Authors:** Ayesha Aftab, Ranjha Khan, Wasim Shah, Muhammad Azhar, Ahsanullah Unar, Hafiz Muhammad Jafar Hussain, Ahmed Waqas

**Affiliations:** 1 Department of Biological Sciences, International Islamic university, Islamabad 44000, Pakistan; 2 The First Affiliated Hospital of USTC, Hefei National Laboratory for Physical Sciences at Microscale, The CAS Key Laboratory of Innate Immunity and Chronic Diseases, School of Life Sciences, CAS Centre for Excellence in Molecular Cell Science, Collaborative Innovation Center of Genetics and Development, University of Science and Technology of China, Hefei 230027, China; 3 Shanghai Jiao Tong University, School of Medicine, Shanghai, China; 4 Department of Zoology, Division of Science and Technology, University of Education Lahore, Multan Campus, Multan, Pakistan

**Keywords:** Breast cancer, CCND1, Cyclin D1, SNPs, Variants

## Abstract

*CCND1* encodes for Cyclin D1 protein and single-nucleotide polymorphisms (SNPs) can modulate its activity. In the present study, the impact of *CCND1* SNPs on structure and/or function of Cyclin D1 protein using *in silico* tools was investigated. Our analysis revealed only one splice site SNP (c.1988+5G<A) can effect *CCND1* function. Subsequently, 78 out of 169 missense variants were predicted as pathogenic by Polyphen2, SIFT, PROVEAN, SNPs&GO, and PANTHER, and 4/78 missense SNPs were further evaluated because these four SNPs were found to be reside in highly conserved region of Cyclin D1. However, they did not show any major impact on tertiary structure and domain of Cyclin D1 but overall R15S and A190S has displayed a significant diseased phenotype and an altered molecular mechanism predicted by MutPred, FATHMM, SNPeffect, SNAP2, and PredictSNP. Consistently, A190S, R179L, and R15S may also cause a decrease in stability of Cyclin D1 anticipated by I-Mutant, HOPE and SNP effect. Furthermore, the Kaplan–Meier plotter has explained that high expression of *CCND1* is associated with less survival rate of breast cancer patients. Altogether our study suggests that c.1988+5G<A, R15S, R179L, and A190S SNPs could directly or indirectly destabilize Cyclin D1.

## Introduction

Breast cancer is a heterogeneous type of cell carcinoma with high rate of morbidity and mortality in women [1]. Since 2008, every year approximately 2 million cases of breast cancer are being diagnosed and approximately 50% cases belonged to developing countries with high rate of mortality [2]. Similarly, due to dwindling resources, lack of high-throughput and innovative technologies to deal the breast cancer management and diagnosis is the major reason of continuously increasing cases of breast cancer in developing countries. On the other hand, it has been reported that breast cancer cases are also going to increase in young women. The evidence has demonstrated that women with age <45 years are facing the leading cause of breast cancer [3]. Thus, despite of emergence of new medical approaches and intensive research, still breast cancer is a major health problem and top priority is given to breast cancer in medical research.

*CCND1* encodes Cyclin D1 protein that is an important regulator of G1 phase of the cell cycle. Generally, Cyclin D1 function in association with its Cyclin-dependent kinase (CDK) partner such as CDK4 and CDK6, thus, mediating phosphorylation and inactivation of retinoblastoma protein [4]. Dysregulation of Cyclin D1 is frequently linked with various type of cancer in human with diverse histological origin, and thus, it is considered a potential biomarker for diagnosing of different cancers [5,6]. Previous studies have demonstrated that Cyclin D1 overexpression is the main cause of cancer due to the splice modulation by a polymorphism, A870G, in the donor region of the exon 4/intron boundary [7]. Recently, A870G, polymorphism have been reported in the oesophageal adenocarcinoma [8]. Subsequently, its dysregulation is also reported in breast cancer and transgenic mice of *CCND1* gene also displayed altered mammary cell proliferation and adenocarcinomas [9]. However, the underlying mechanism of Cyclin D1 role in breast cancer is still unknown.

SNPs occur once in every 1000 nucleotides and are positioned in the DNA between genes which are acting as biological marker to locate the genes that are associated with disease. But these SNPs, when occurs within the gene or in regulatory region, they may cause the onset of complex diseases like diabetes and cancer [10]. The Cyclin D1 is found to be involve in complex network signalling with other proteins and forms a *CCND1–*CDK4 complex (DC) with CDK4. The variations in *CCND1* might cause a change in its transcript or translational yield. Therefore, in the present study SNPs of *CCND1* were selected on basis of minor allele frequency (MAF) ranging from 0.0001 to 0.05 and computationally analysed in order to predict their impact on Cyclin D1 function and to evaluate their role in breast cancer.

## Methodology

### Dataset collection

**Figure 1:**
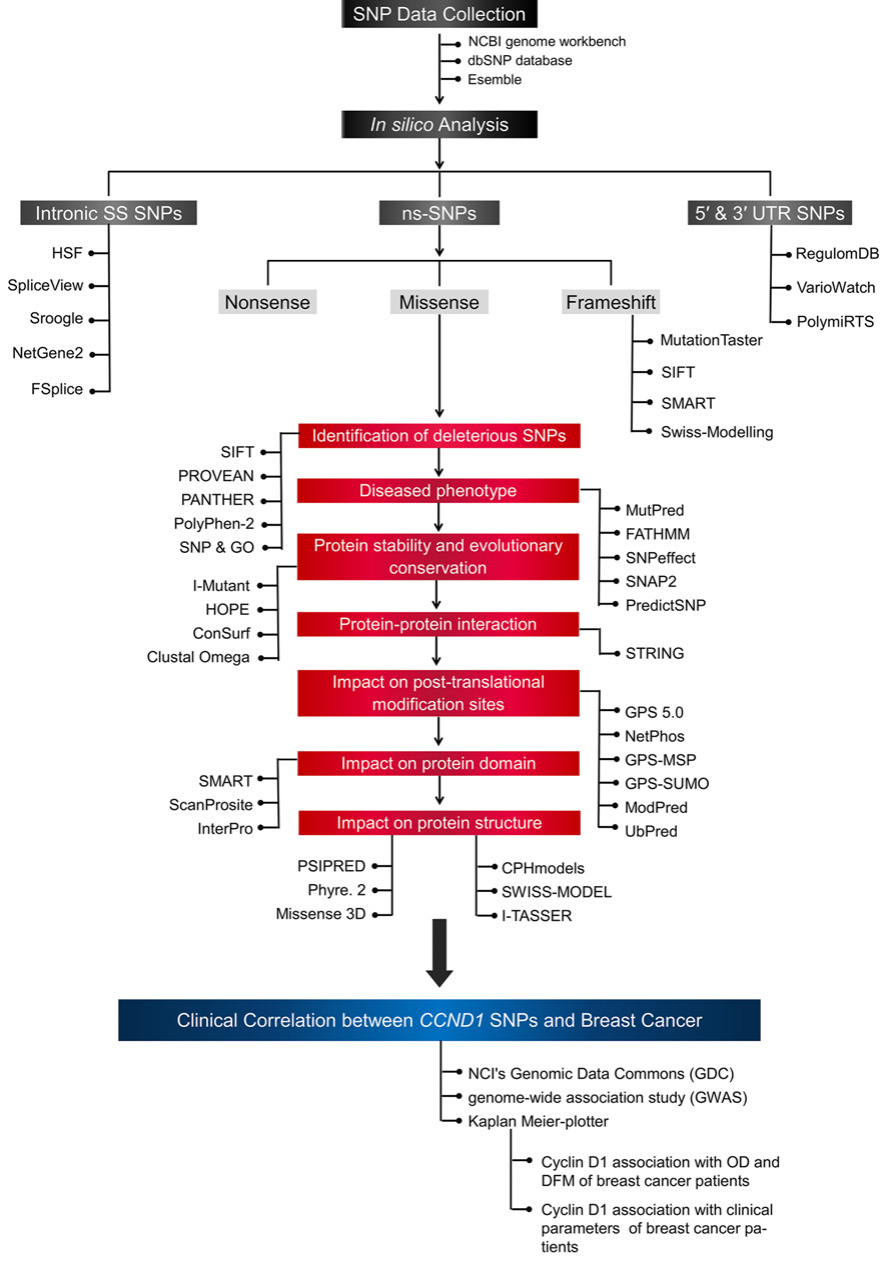
The flow chart of *in silico* analysis steps taken to predict the impact of *CCND1* SNPs on its protein structure and function, and clinical correlation Abbreviations; DFM, disease-free survival; OS, overall survival; SS, splice site.

The *CCND1* SNPs were collected from National Centre for Biotechnology Information (NCBI) genome workbench (https://www.ncbi.nlm.nih.gov/tools/gbench/) and Single Nucleotide Polymorphism Database (dbSNP) of NCBI (https://www.ncbi.nlm.nih.gov/snp). The SNP data of splice site, non-synonymous SNP, 3´ UTR and 5´ UTR SNPs were selected for *in silico* analysis. The details flow chart of steps followed in the present study is given in Figure 1. The nucleotide sequence of *CCND1* and amino acid sequence were downloaded from NCBI (https://www.ncbi.nlm.nih.gov/) and ensemble genome browser (https://asia.ensembl.org/index.html), respectively. The UniProt identifier of Cyclin D1 is P24385 and RSC PDB ID is 2w96.A.

### *In silico* analysis of splice site SNP

Splice site SNPs were selected by considering 10 nucleotides at 5´ and 3´ end of intron. The impact of SNPs on splicing was evaluated by recruiting the five online tools for intronic and splice region mutation. These include the HSF (Human Splicing Finder, http://www.umd.be/HSF3/), SpliceView (http://bioinfo.itb.cnr.it/∼webgene/wwwspliceview.html), Sroogle (http://sroogle.tau.ac.il/), Netgene2 (http://www.cbs.dtu.dk/services/NetGene2/), and FSplice v.01 (http://www.softberry.com/berry.phtml?topic=fsplice&group=programs&subgroup=gfind).

### *In silico* analysis of missense variants

The deleterious effect of missense SNPs was analysed using five bioinformatics tools: PolyPhen-2 (Polymorphism Phenotyping v2, http://genetics.bwh.harvard.edu/pph2/), SIFT (Sorting Intolerant from Tolerant, https://sift.bii.a-star.edu.sg/), PROVEAN (Protein Variation Effect Analyzer, http://provean.jcvi.org/index.php), SNP&GO (https://snps.biofold.org/snps-and-go/snps-and-go.html) and PANTHER (http://www.pantherdb.org/tools/). The pathogenic SNPs were selected and additionally filtered and picked on the basis of MAF range 0.0001–0.5, SNP conservation, impact of SNP on protein domain, stability, structure, protein–protein interaction, disease phenotype and post-translational effect. Following tools were accessed for further *in silico* analysis.

**MutPred** (http://mutpred.mutdb.org/) predicts the pathogenicity of amino acid change and the molecular mechanism. It uses Random Forest to predict the *g*-score and *P* values to predict the deleterious mutation. The SNP with *g* score greater the 0.5 are showing the probability of being a deleterious mutation or disease associated. The SNPs are further categorise according to relevant hypotheses. (A) Actionable hypotheses SNP = *g* > 0.5 and *P*<0.05, (B) Confident hypotheses SNP = *g* > 0.75 and *P*<0.05, and (C) Very confident hypotheses SNP = *g* > 0.75 and *P* < 0.01.

**FATHMM** (Functional Analysis through Hidden Markov Models) v2.3 (http://fathmm.biocompute.org.uk/) is used to predict the functional impact of coding and non-coding variants. CScape option was selected to predict the oncogenic status of four deleterious mutations. The input was given in the form of list having chromosome number, position of variants, and mutant.

**SNPeffect database v.4.0** (http://snpeffect.switchlab.org/) was also used to predict the molecular phenotypic impacts SNPs. This database focuses on the effect of mutation on aggregation propensity using TANGO tool, amyloid propensity using WALTZ tool and chaperone binding by using LIMBO tool. It also calculates the effect of mutation on structural stability of protein using FoldX. The input was given as UniProt ID P24385 and corresponding mutation.

**SNAP2** (https://rostlab.org/services/snap2web/) is a neural network that distinguishes the effect and neutral SNP by considering the evolutionary conservation, secondary structure, and solvent accessibility effect caused by SNP. The output score ranges from -100 to +100 predicting the strong neutral prediction to strong diseased effect, respectively.

**PredictSNP v.2** (https://loschmidt.chemi.muni.cz/predictsnp2/) is a powerful tool that identifies the functional impact of SNP by utilizing six databases CADD, DANN, FATHMM, FitCons, FunSeq2, and GWAVA to develop category-optimal decision thresholds. The output displays the results of five best performing tools in form of neutral, deleterious, and unknown.

**HOPE** (Have (y)Our Protein Explained, https://www3.cmbi.umcn.nl/hope/) is a next-generation web application for automatic mutant analysis. HOPE combines the information from UniProt, Reprof, and PDB to analyse the effect of mutation on protein structure.

**I-Mutant 2.0** (http://folding.biofold.org/i-mutant/i-mutant2.0.html) is a tool used for prediction of protein stability upon single site mutation. The data set of the tool is resultant from ProTherm which is the most comprehensive database of protein mutation. It predicts the reliability index (RI) ranging from 0 to 10, where 10 is the highest reliability, the DDG in kcal/mol which is the free energy change value [11]. The I-Mutant query was set at 25°C and pH7.

**STRING** (https://string-db.org/) is a large database of known and predicted protein–protein signalling interactions. The output is given in form of nodes and edges that represent proteins and interaction, respectively. The output scores are indicators of confidence, i.e. how likely STRING judges an interaction to be true, given the available evidence. Instead, they are in order to predict the possible interaction of Cyclin D1 with other proteins; the input name was given as *CCND1* and *Homo sapiens* was selected as organism.

**ConSurf Sever** (https://consurf.tau.ac.il/) estimates and visualizes evolutionary conservation in macromolecules. The WT amino acid sequence was given as input, HMMER was selected as homology search algorithm, and calculations were based on Bayesian method. According to this method, the conservation score ranges from 1 to 9, the 1–4 is assigned as variable residue, 5–6 as average, and 7–9 as highly conserve residues.

**Clustal Omega** (https://www.ebi.ac.uk/Tools/msa/clustalo/) is MSA program for alignment between three or more sequences. The amino acid sequence of human Cyclin D1 (UniProt: P24385) and 19 other species was downloaded, and alignment was performed using online Clustal Omega server.

#### Prediction of post-translational modifications sites and effect of mutation on it

Post-translational modifications are covalent modifications which modify the protein structure to play an essential role in cellular signalling pathways and networks. For this purpose, we access the easy-to-use CUCKOO Workgroup (http://www.biocuckoo.org/) consisted of several web tools. GPS 5.0 (Group based prediction system), GPS-SUMO, and GPS-MSP (Methyl-group Specific Predictor), BDM-PUB (http://bdmpub.biocuckoo.org/index.php) for prediction of phosphorylation, sumoylation methylation, and ubiquitilation, respectively. In future, we have predicted the post-translational modification sites in WT and effect of SNP by accessing the NetPhos (http://www.cbs.dtu.dk/services/NetPhos/), ModPred (http://www.modpred.org/) for potential phosphorylation, methylation, sumoylation, and ubiquitilation. Ubpred (http://www.ubpred.org/) was also used for prediction of potential ubiquitination sites in Cyclin D1. To access these tools, the amino acid sequence of Cyclin D1 WT and mutant was submitted in FASTA format.

#### SNPs impact on Cyclin D1 domains

The impact of SNPs on Cyclin D1 domain was predicted SMART (http://smart.embl-heidelberg.de/), ScanProsite (https://prosite.expasy.org/scanprosite/), and InterPro (https://www.ebi.ac.uk/interpro/). InterPro is an integrated database that predicts and displays the results from different databases like SMART, Prosite, Conserved Domains Database (CCD), and Pfam protein domain database. The input of SNPs in all these tools was given in the form of FASTA format, while WT/ Cyclin D1 domain analysis was done by giving its UniProt ID. The output of WT and mutant protein was compare in order to predict the change caused by SNP. The description of each binding site was taken from InterPro database (https://www.ebi.ac.uk/interpro/).

#### Secondary structure prediction and structural homology modelling

The effect of SNP on secondary structure of the Cyclin D1 was analysed using PSIPRED (http://bioinf.cs.ucl.ac.uk/psipred/). Further, Missense3D (http://www.sbg.bio.ic.ac.uk/∼missense3d/) was used that matches or compare the WT structure of protein with its mutant and provide detail output of change. Phyre2 (Protein Homology/analogY Recognition Engine V 2.0, http://www.sbg.bio.ic.ac.uk/phyre2/html/page.cgi?id=index) predict the secondary structure of the WT protein and mutant protein by providing the FASTA format of the amino acid sequence in input. The homology modelling of the Cyclin D1 and its mutant protein was performed using the SWISS-MODEL (https://swissmodel.expasy.org/), CPHmodels 3.2 server (http://www.cbs.dtu.dk/services/CPHmodels/), and confirmed by I-TASSER (Iterative Threading ASSEmbly Refinement) models (https://zhanglab.ccmb.med.umich.edu/I-TASSER/). The structures were visualized and analysed by using Swiss Pdb-Viewer v.4.1.

### Frameshift SNPs impact on Cyclin D1 structure and function

*CCND1* frameshift mutations were also filtered on the basis of MAF and *in silico* investigation was done to study their pathogenic effect. MutationTaster (http://www.mutationtaster.org/) was accessed to predict the disease-causing potential effect of an SNP. The impact of SNPs on protein structure and protein domain was predicted by SWISS-MODEL and SMART, respectively. Furthermore, the SIFT *in silico* analysis was performed to verify the pathogenic effect and also the probability of occurrence of non-sense mediated decay (NMD).

### Effect of 3′ and 5′ UTR SNPs on Cyclin D1

The 3′ and 5′ UTR SNPs were also selected on the basis of MAF ranges. The effect of SNPs on regulatory units were explored by using multiple *in silico* tools, i.e. RegulomDB (https://regulomedb.org/regulome-search/), Variowatch (http://grch38.genepipe.ncgm.sinica.edu.tw/variowatch/main.do), and PolymiRTS (http://compbio.uthsc.edu/miRSNP/miRSNP_detail_all.php). RegulomDB is a database that predicts regulatory elements in intergenic regions [12]. Variowatch is an automatic data mining tool retrieving genomic information about an SNPs and provides a risk level on functional impact from very low to very high of genomic variants [13]. The PolymiRTS is a database for identified mirSNPs allowing also the evaluation of SNP from dbSNP that classify DNA polymorphism in target sites of miRNA and miRNAs and explain their links physiological behavioural, molecular and disease phenotypes [14].

### Clinical association of *CCND1* SNPs

The SNPs which damage the structure and function of a protein may lead to onset of a disease. The *CCND1* SNPs that has shown a clinical association were sorted out using NCBI. The data mining at The NCI’s Genomic Data Commons (GDC, https://portal.gdc.cancer.gov/) was performed. GCD is a cancer data repository that provides cancer genomic studies to support precision medicines. We have also performed a comprehensive search to collect the SNPs of *CCND1* that are associated with breast cancer by genome-wide association study (GWAS, https://www.ebi.ac.uk/gwas/). Furthermore, the clinical significance of *CCND1* was related with the survival of breast cancer patients using the Kaplan–Meier plotter (https://kmplot.com/analysis/). Kaplan–Meier plotter has assessed of 54,000 genes to evaluate their expression with survival in 21 cancer types and has the data of over 10,000 samples of patients with cancer, out of which 6234 are patients with breast cancer. The source of this system include Gene Expression Omnibus (GEO), European Genome-Phenome Archive (EGA), and The Cancer Genome Atlas (TCGA). In this research study, the *CCND1* expression was also associated with overall survival (OS), disease-free survival (DFS), and clinical parameters of patients with breast cancer.

## Results

**Figure 2:**
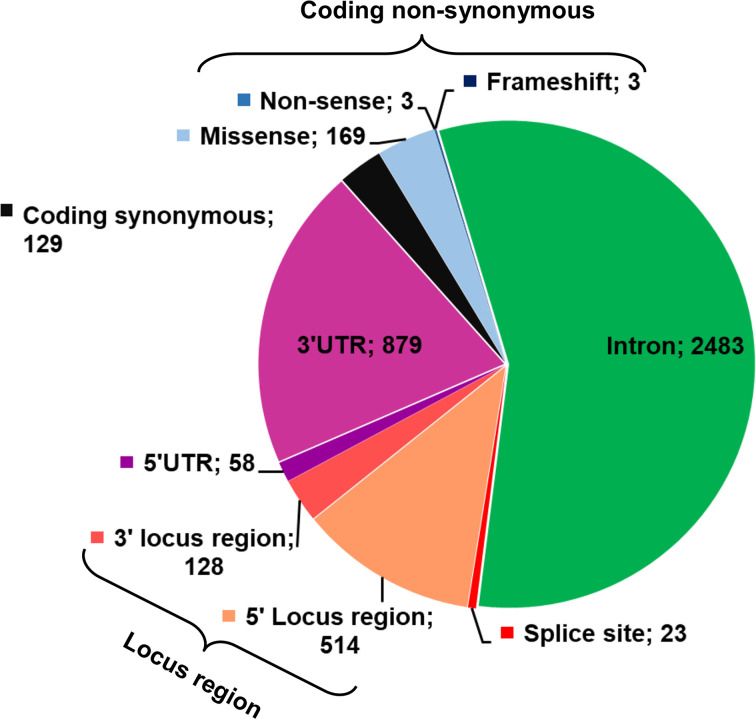
The pie chart displaying the total number of different SNPs of *CCND1*

The *CCND1* SNP data were collected from the NCBI genome workbench. Total 3747 *CCND1* SNPs were collected which were consisted of coding non-synonymous SNPs (non-sense, miss-sense and frameshift), coding synonymous SNPs, SNPs in 5′ and 3′ UTR, 3′ and 5′ locus region SNPs and non-coding SNPs (intron and splice site region). The number of different functional classes of SNPs is given in Figure 2. In this research study, the *in silico* analysis of coding non-synonymous SNPs, splice site SNPs and 5′ and 3′ UTR SNP was carried out to predict their pathogenic impact on protein structure and function (Figure 1).

### *In silico* analysis of splice site SNP

The mutation in splicing region causes the improper exon and intron recognition by splicing machinery which results in an aberrant transcript. The variants residing within 10 nucleotide position in intronic splice site region account more in creating defective splice site [15]. In this research, 23 SNPs out of total 2506 intronic SNPs were collected which occurs within 10 nucleotide position at 5′ and 3′ region of intron. The *in silico* analysis of 23 splice site SNPs was performed by HSF, Sroogle, SliceView, NetGene2 and FSplice comparing the *CCND1* WT and mutant scores. The *in silico* analysis of these tools has predicted that the SNP can affect the donor or acceptor site of splice region (Supplementary Table S1). Out of these 23 SNPS, only one SNP (rs752676953, c.1988+5G<A) was found to be probably damaging by four *in silico* tools, the HSF, Sroogle, NetGene2 and FSplice, given in Table 1. The Netgene2 and FSplice tools have predicted that rs752676953 forms a mutated region that may not be recognized as splice site by the splice site machinery and results in exon skipping which lead to alerted protein function.

**Table 1:** *CCND1* intronic splice site SNP effecting splicing predicted by *in silico* analysis

RS ID	SNP	HSF*matrix score (0–100)				SpliceView score (0–100)		Sroogle			NetGene2 confidence score (0-1)		FSplice (weight index)	
		WT	Mu	Variation %	Interpretation	WT	Mu	Element	WT	Mu	WT	Mu	WT	Mu
rs752676953	g.5412G>A, c.198+5G>A	86.34	74.18	-14.08	Probably effecting splicing	84 DS	78 DS	5′ SS	-3.80 (Delta-G) 6.79 (Max entropy)	-3.80 (Delta-G) 2.02(Max entropy)	0.70 DS	Not generated	W = 7.36	Not generated

* HSF, Human Splicing Finder

### Identification of pathogenic missense variants from Cyclin D1 SNPs pool

Out of 169 missense variants, 78 were predicted to be deleterious (Supplementary Table S2). These 79 were further sorted out on basis of their MAF range (0.0001–0.05) and only 4 SNPs (rs557545630, rs534553548. rs535957987, rs143479406) were shortlisted (Table 2) and computationally evaluated for their impact on protein structure and function using different *in silico* tools.

**Table 2:** Highly deleterious missense substitution having MAF range 0.0001–0.5

RS ID	Missense substitutions	PolyPhen-2*		SIFT^†^		PROVEAN^‡^		SNP&GO^§^		PANTHER^║^	
		Scores	Prediction	Scores (0-1)	Prediction	Score	Prediction	Probability	Prediction	Probability	Prediction
rs557545630	NM_053056.2:c.43C>A, NP_444284.1:p.Arg15Ser	0.994	Probably Damaging	0.01	Damaging	-3.8	Deleterious	0.957	Disease	0.361	Neutral
rs534553548	NM_053056.2:c.568G>T, NP_444284.1:p.Ala190Ser	0.894	Possibly damaging	0.12	Tolerant	-2.2	Neutral	0.942	Disease	0.635	Disease
rs535957987	NM_053056.2:c.876C>G, NP_444284.1:p.Asp292Glu	0.994	Probably Damaging	0.15	Tolerant	-2.56	Deleterious	0.785	Disease	0.257	Neutral
rs143479406	NM_053056.2:c.536G>A, NP_444284.1:p.Arg179His,	0.919	Possibly damaging	0.05	Tolerant	-3.84	Deleterious	0.907	Disease	NA	Unclassified
	NM_053056.2:c.536G>T, NP_444284.1:p.Arg179Leu	0.947	Possibly damaging	0.02	Damaging	-5.10	Deleterious	0.837	Disease	0.268	Neutral

* PolyPhen-2 = Polymorphism Phenotyping v2, Scores near to 1 are more confidently predicting the SNP to be damaging. http://genetics.bwh.harvard.edu/pph2/

† SIFT = Sorting Intolerant From Tolerant, SNP scores near 0.00 are more predicted as damaging. http://sift.bii.a-star.edu.sg/

‡ PROVEAN = Protein Variation Effect Analyzer, cut off -2.5, scores equal to or above this threshold are predicting the SNP as deleterious http://provean.jcvi.org/index.php

§ SNP&GO = scores equal or above 0.5 are predicting the SNP as diseased http://snps.biofold.org/snps-and-go/snps-and-go.html

║ PANTHER = PANTHER scores are given along with SNP&GO scores, scores equal or above 0.5 are predicting the SNP as diseased http://snps.biofold.org/snps-and-go/snps-and-go.html

#### Disease phenotype and molecular mechanism associated with SNPs

MutPred was used to analyse the four selected missense SNPs for prediction of their probability of damaging the protein and molecular mechanism that they can alter (Table 3). It was found that R15S, D292E, and R179L were damaging for protein structure as their *g* score were greater than 0.5. Furthermore, the R15S and D292E were predicted to undergo the molecular changes. R15S has altered the metal binding pocket of Cyclin D1, while D292E has more damaging action that is causing the loss of relative solvent accessibility, the gain of strand and also has altered the metal binding property. The results from FATHMM analysis has shown a low-confidence oncogenic predictions of SNP R15S (*P*=0.523777), A190S (*P*=0.740342), R179H (*P*=0.875302), and R179L (*P*=0.859033) and association with cancer because the *P* values above 0.5 are predicted to be deleterious. However, D292E (*P*=0.488885) was predicted to be low-confidence neutral and it may not be associated with cancer. Consistently, the web server SNPeffect results (Supplementary Table S3) has predicted that A190S decreases the aggregation tendency of Cyclin D1 and this SNP results in a Δ*G* of 1.15 kcal/mol which implies that the mutation has reduces the protein stability. While other SNPs have no effect on aggregation tendency, amyloid propensity and chaperone binding tendency of Cyclin D1. We have also included the SNAP2 prediction results to evaluate the most disease causing SNP. The SNAP2 has predicted R15S and D292 as ‘effect’ causing SNPs and their scores demonstrates their ’medium’ effect for causing disease phenotype. In order to further validate the effect of SNPs, the PredictSNP analysis was performed and its results displayed that all SNPs are leading towards the disease expect D292E as described in Table 4.

**Table 3:** MutPred analysis and prediction of diseased phenotype and molecular mechanism of SNPs

SNP rs ID	Amino acid change	Molecular mechanism	MutPred2 score/ *g* scores	*P*-value	Interpretation*
rs557545630	Arg15Ser	Altered metal binding	0.744	0.03	Disease associated actionable hypotheses
rs534553548	Ala190Ser	NP	0.399		Neutral
rs535957987	Asp292Glu	Loss of relative solvent accessibility	0.753	7.2e-03	Disease associated
		Gain of Strand		0.04	Confident hypotheses
		Altered metal binding		0.01	Confident hypotheses
rs143479406	Arg179His,	NP	0.468	NP	Neutral
	Arg179Leu	NP	0.652	NP	Disease associated

Threshold *P* value ≤ 0.05, NP = not predicted

* Interpretation is done on basis of *g* and *P* score; actionable hypotheses: *g* > 0.5, *P*<0.05; confident hypotheses: *g* > 0.75, *P*<0.05 and very confident hypotheses: *g* > 0.75, *P*<0.01. Disease associated when *g* > 0.5

**Table 4:** PredictSNP analysis results for diseased phenotype

SNP rs ID and amino acid change	Tools and their prediction						Possible impact*
	PredictSNP	CADD	DANN	FATHMM	FunSeq2	GWAVA	
rs557545630 R15S	Neutral	Deleterious	Deleterious	Deleterious	Deleterious	Deleterious	Diseased
rs534553548 A190S	Deleterious	Deleterious	Deleterious	Deleterious	Deleterious	Deleterious	Diseased
rs535957987 D292E	Neutral	Neutral	Neutral	Neutral	Deleterious	Neutral	Neutral
rs143479406 R179H	Deleterious	Deleterious	Deleterious	Deleterious	Deleterious	Deleterious	Diseased
rs143479406 R179H	Deleterious	Deleterious	Deleterious	Deleterious	Deleterious	Deleterious	Diseased

* SNPs predicted as damaging by four or more tools are classified as diseased.

Interestingly, the overall predictions from MutPred, FTHMM, SNP effect, and SNAP2 are not in match with each other; this may be due to low confidence score of SNP in favour of its effect as diseased. However, according to these results and PredictSNP prediction, R15S and A190S may be predicted to cause a more diseased effect with change in the molecular mechanism.

#### High risk changes in protein stability and evolutionary conservation

The amino acid that are most critical to the protein structure and stability are found to be evolutionary conserved. In order to predict the stability of protein, I-Mutant was accessed. I-Mutant has predicted that all the SNPs caused decreased protein stability except one SNP that is D292E (Table 5). The highest instability found was −0.45 kcal/mol energy related to R179L SNP. Furthermore, HOPE was run to analyse the impact of four SNPs on conservation and protein structure. HOPE has predicted two SNPs (A190S and R179H) that might be highly damaging to the Cyclin D1 protein Table 6. These SNPs can impact the incorrect new bonding and may results in alternation of Cyclin D1 function. The results from SNPeffect has also predicted the instability of Cyclin D1 due to A190S SNP.

**Table 5:** Prediction of protein stability using IMutant v.2.0

SNP rs ID	Amino acid change	Stability	RI (0-10)*	DDG (Kcal/mol)*
rs557545630	R15S	Decreased	9	-2.51
rs534553548	A190S	Decreased	9	-0.40
rs535957987	D292E	Increase	3	0.29
rs143479406	R179H	Decreased	8	-1.28
	R179L	Decreased	4	-0.45

* IMutant analysis was accessed at temperature 25°C and pH 7. The reliability index (RI) range is 0–10, where 10 is highest index number, DDG is free energy change value. DDG<0: Decrease Stability and DDG>0: Increase Stability

**Table 6:** Prediction of change in structure, domain and conservation of Cyclin D1 du to missense SNP using HOPE *in silico* analysis

RS ID	Schematic structure	Amino acid properties	Structure	Domain (InterPro)	Conservation
rs557545630 R15S	> 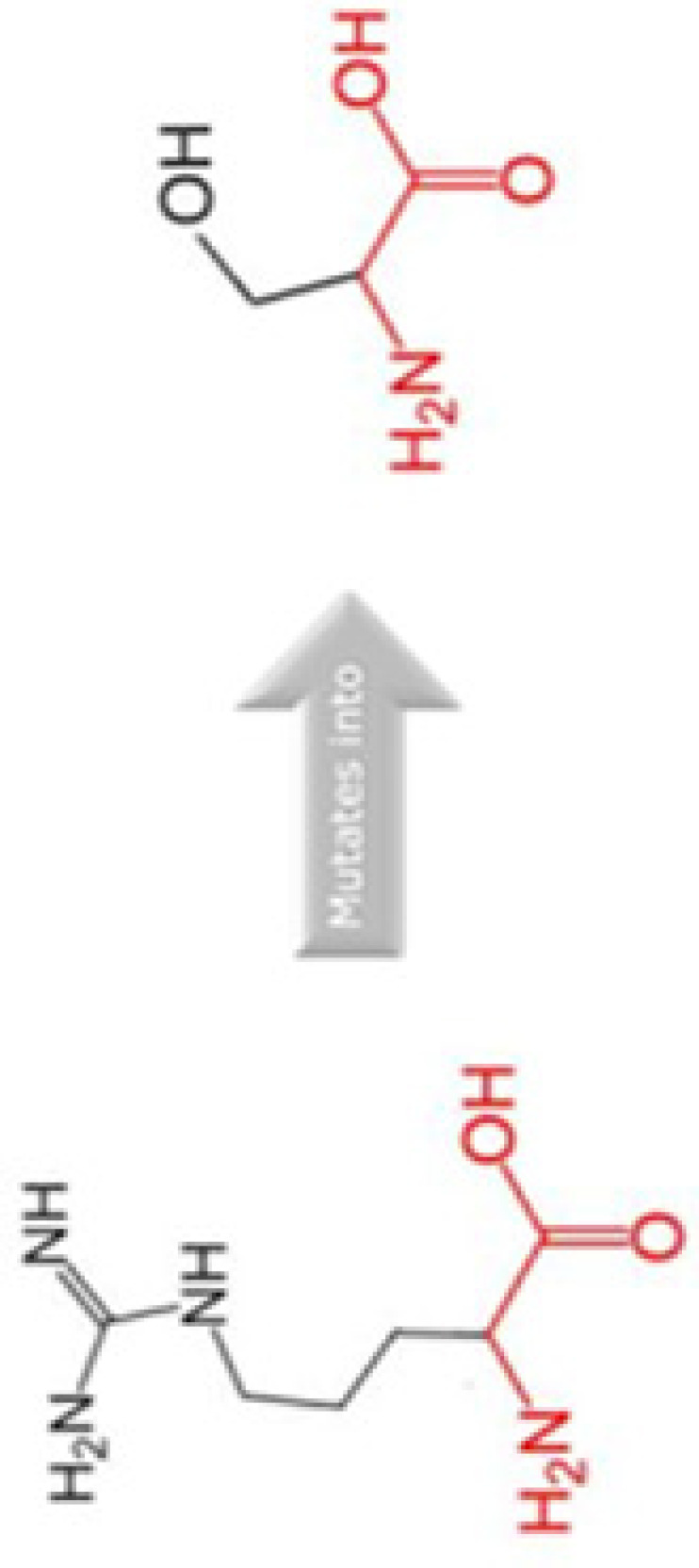	The mutant residue is smaller than the WT residue, and might lead to loss of interactions. The WT residue charge was POSITIVE; the mutant residue charge is NEUTRAL, and can cause loss of interactions with other molecules or residues. The mutant residue is more hydrophobic than the WT residue, and can result in loss of hydrogen bonds and/or disturb correct folding.	No impact on structure of protein predicted	No impact on domain was predicted	The mutant residue is located near a highly conserved position and have some properties in common with WT mutated residue. This means that in some rare cases this mutation might occur without damaging the protein.
rs534553548 A190S	> 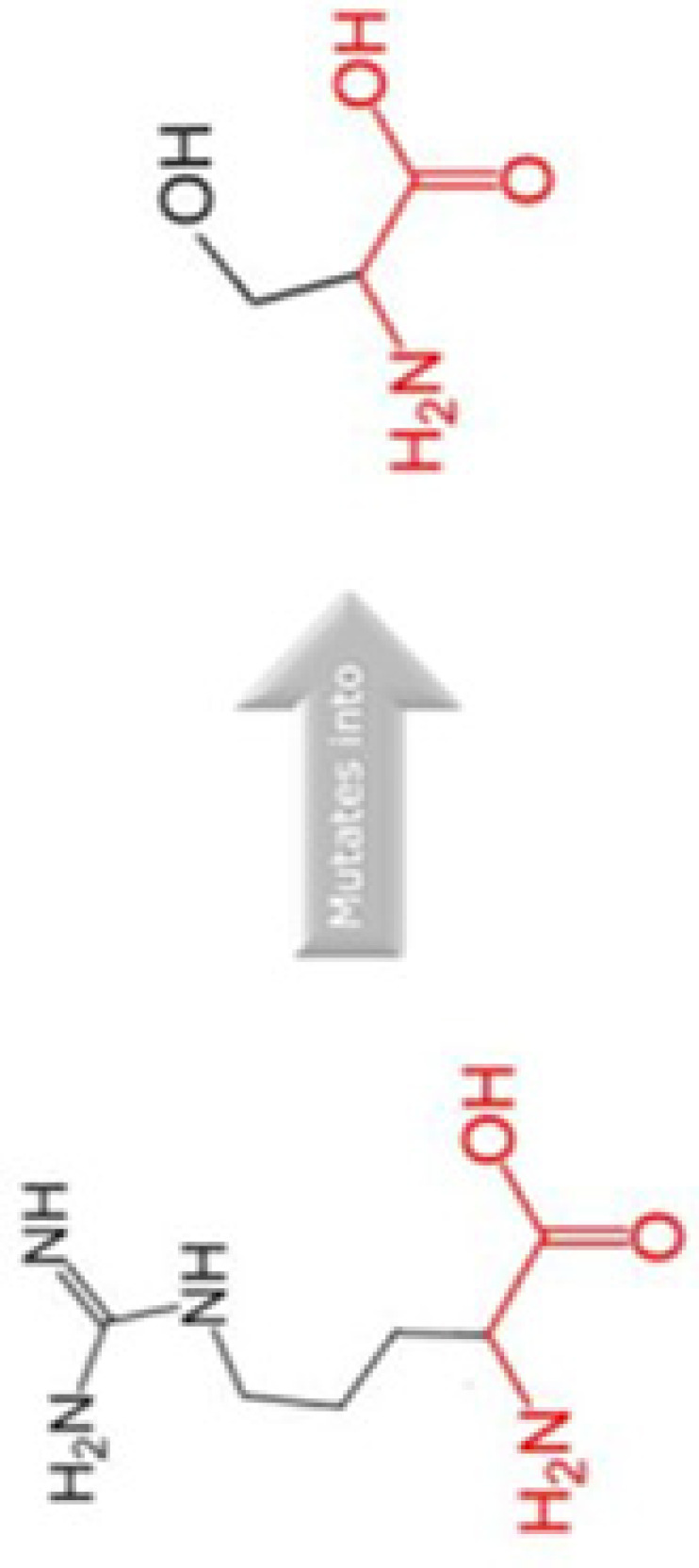	The mutant residue is bigger than the WT residue and probably will not fit in core of protein. The hydrophobicity of the WT and mutant residue differs. The mutation will cause loss of hydrophobic interactions in the core of the protein.	In the 3D-structure, it can be seen that the WT residue is located in an α-helix. The mutation converts the WT residue in a residue that does not prefer α-helices as secondary structure.	The residue is buried in the core of a domain. The differences between the WT and mutant residue might disturb the core structure of this domain.	Mutant residue is located near a highly conserved position. This mutation might occur in some rare cases, but it’s more likely that the mutation is damaging to the protein.
rs535957987 D292E	> 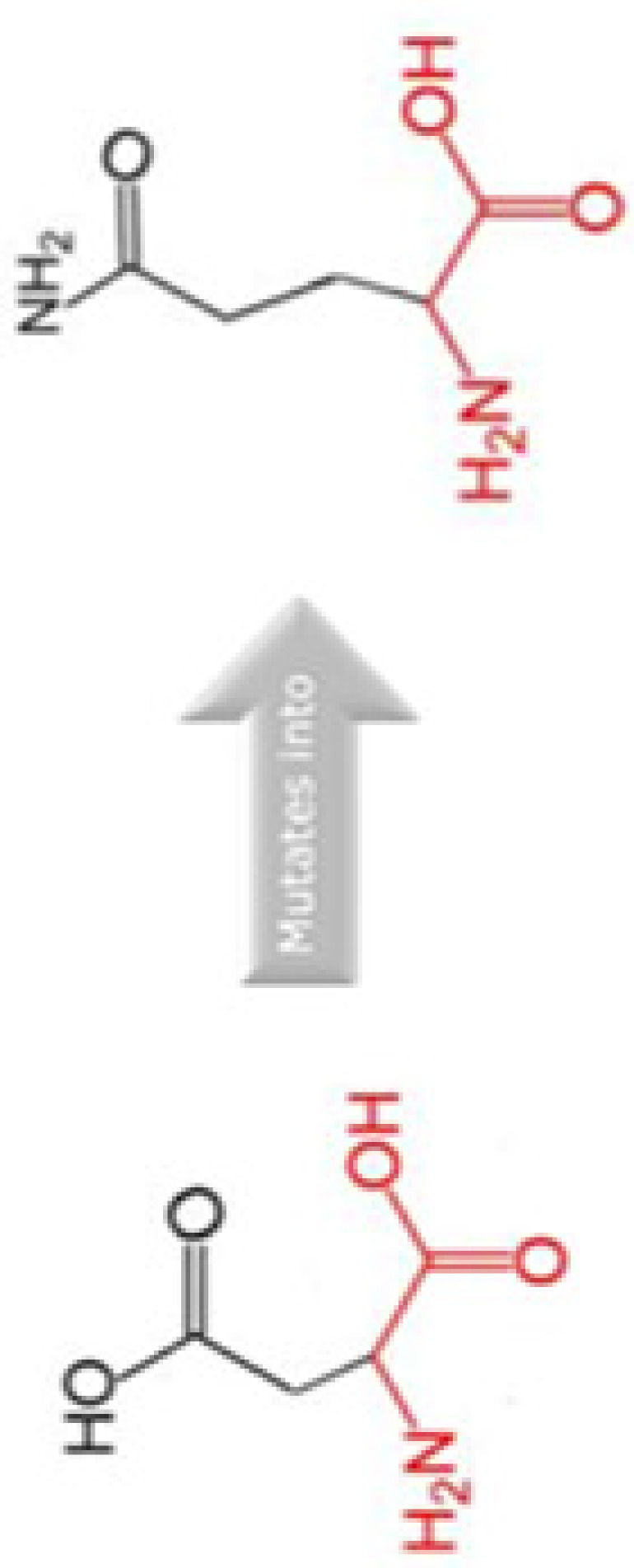	The WT residue charge was NEGATIVE, the mutant residue charge is NEUTRAL, which may cause loss of interactions with other molecules or residues. The mutant residue is bigger, this might lead to bumps.	No impact on structure of protein predicted.	No impact on domain was predicted.	The mutant residue was not among the other residue types observed at this position in other, homologous proteins. However, residues that have some properties in common with mutated residue were observed. This means that in some rare cases this mutation might occur without damaging the protein.
rs143479406 R179H	> 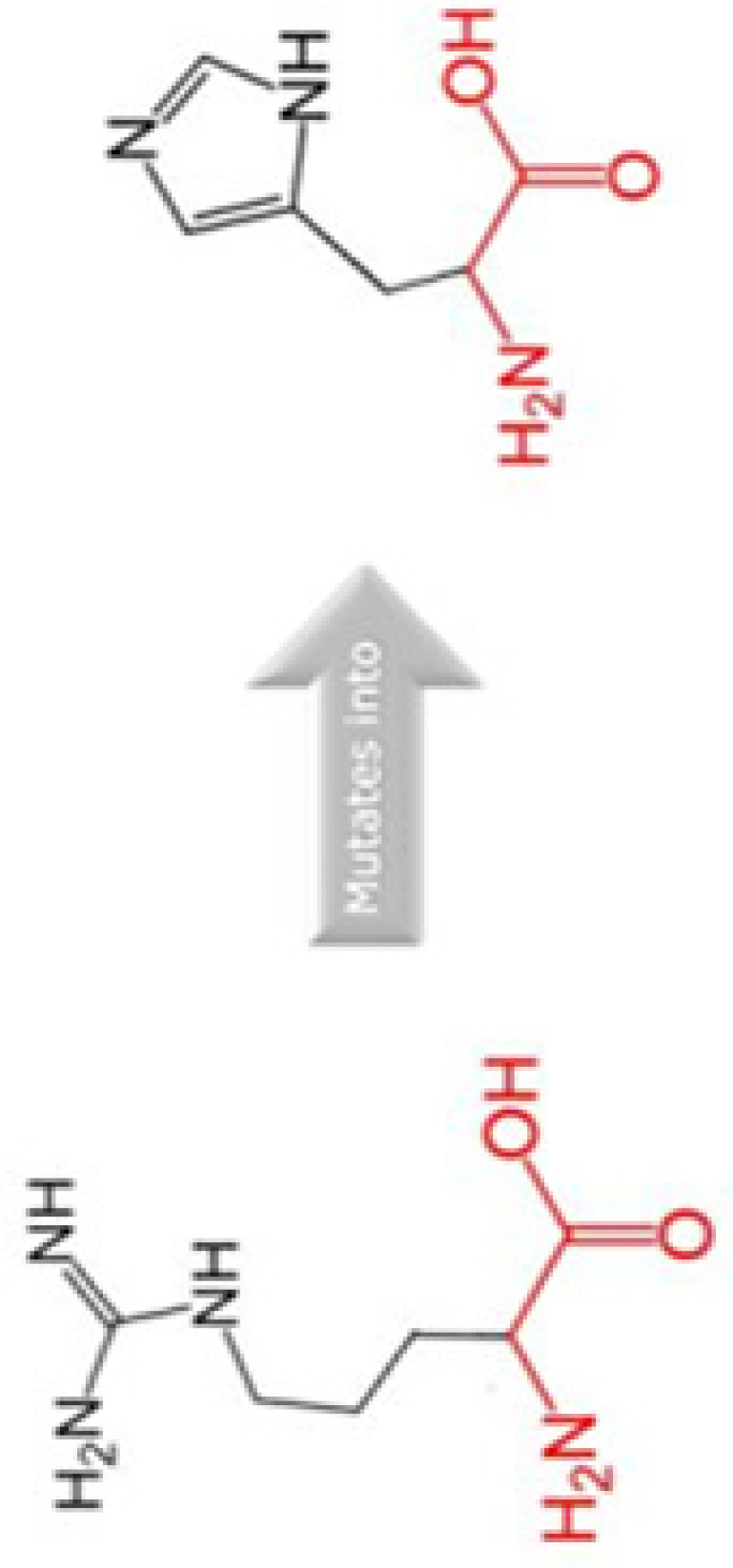	The WT residue charge was POSITIVE; the mutant residue charge is NEUTRAL. Which may cause loss of interactions with other molecules or residues. The mutant residue is smaller than the WT residue. This will cause a possible loss of external interactions.	The WT residue forms a hydrogen bond with glutamic acid at position 162 and Glutamine at position 176, and salt bridge with glutamic acid at position 162, glutamic acid at position 172. The size difference will not makes that the new residue in the correct position to make the same hydrogen bond and salt bridges.	The mutated residue is located on the surface of a domain with unknown function. The residue was not found to be in contact with other domains of which the function is known within the used structure. However, contact with other molecules or domains is still possible and might be affected by this mutation.	The mutant residue is located near a highly conserved position. Neither this mutant residue nor another residue type with similar properties was observed at this position in other homologous sequences. Based on conservation scores this mutation is probably damaging to the protein.

**Figure 3:**
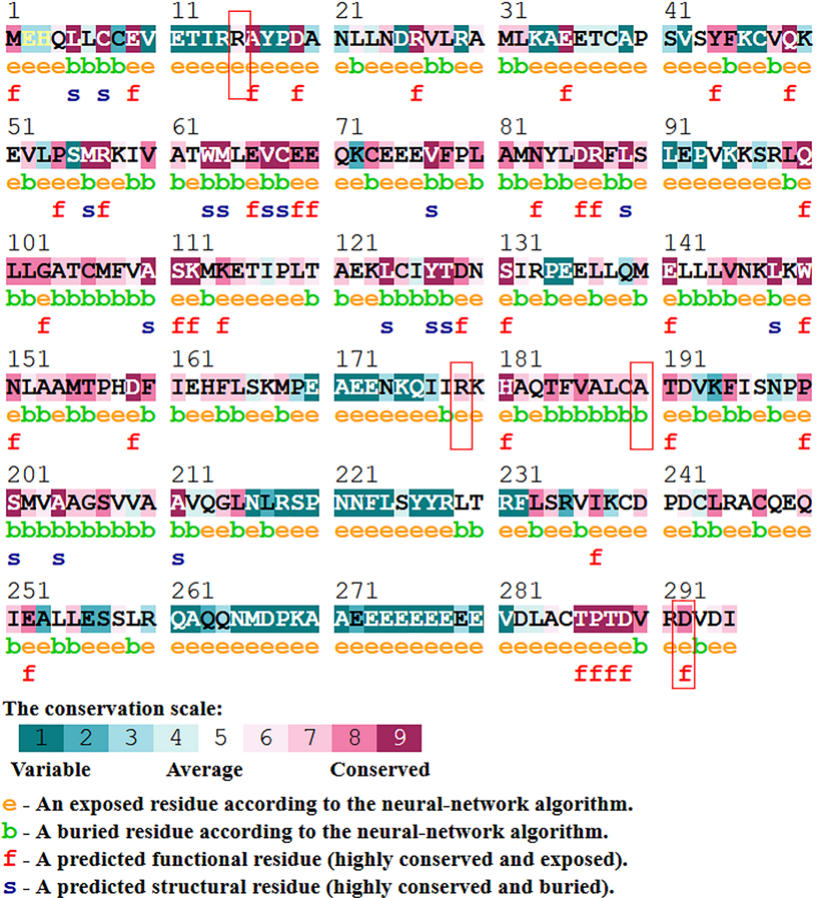
Evolutionary conservation and functional residue prediction by ConSurf

The evolutionary conservation of Cyclin D1 amino acid predicted by ConSurf is shown in Figure 3. The SNP position in WT sequence is shown with red-outlined boxes. The amino acid D292 is predicted to be exposed and highly conserved, i.e. a functional residue. Residue R15 and R179 predicted to be exposed while A190 is buried residue. Residue R179 and A190 are also found to be conserved. Any SNP occur in this region may cause damage to the stability and structure of Cyclin D1. We have also performed MSA of human Cyclin D1 with 20 different organisms using Clustal Omega in order to validate the conservation of these residues, the MSA id given in Supplementary File S1, which predicts that R15, R179, A190, and D292 amino acids are highly conserved, any change in amino acid may have a damaging effect on protein structure and its function. Thus, it can be inferred that these missense mutations are conserved in nature, any alternation on their position can lead to unstable protein structure.

#### Prediction of protein–protein interaction

**Figure 4:**
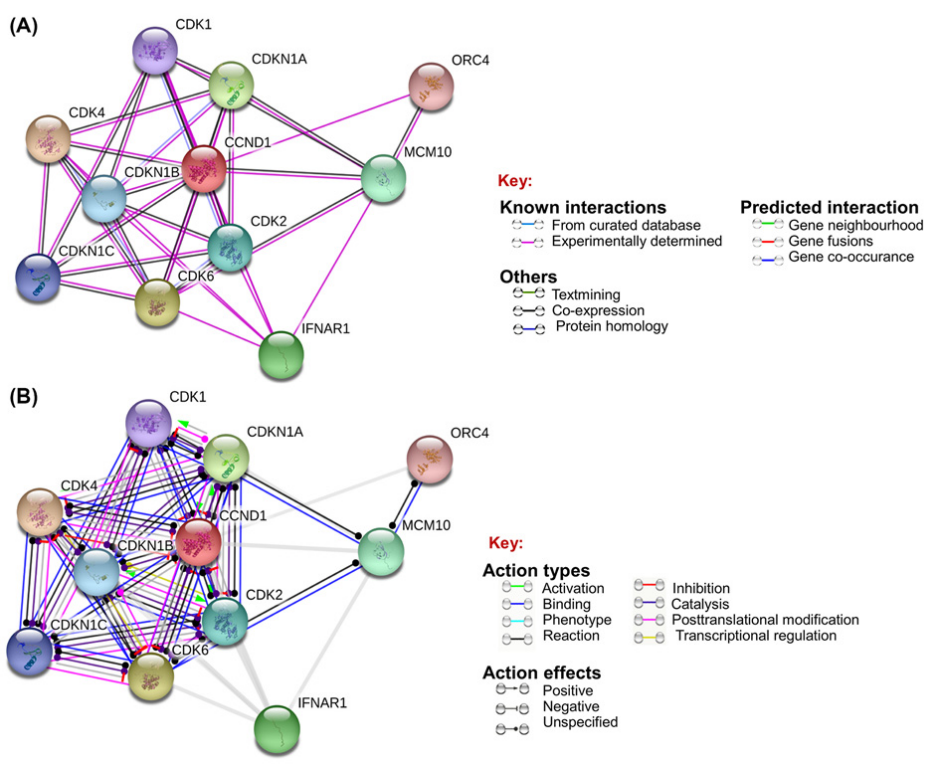
Prediction of protein–protein interaction using STRING v.11.0 (**A**) The interaction evidence and (**B**) molecular action of *CCND1* protein (Cyclin D1) with other proteins.

The Figure 4 and Table 7 indicate the interaction evidence and molecular action of *CCND1* protein (Cyclin D1) with other proteins using STRING. These proteins are meant to be jointly contribute to a shared function with Cyclin D1. The *CCND1* protein is found in strong network signalling with CDK1/2/4/6, CDKN1A/B and in indirect association with MCM10, ORC4, and IFNAR1. In molecular interaction analysis Cyclin D1 is predicted to be directly involve in binding, reaction, catalysis, and inhibition. It is also involving in post-translational modification in association with CDK4/6. Out of these proteins, the CDK4 is a regulatory component of the DC complex which is a major integrator of various mitogenic and antimitogenic signals, and plays a vital role in cancer. The STRING analysis shows that Cyclin D1 is in strong association with the proteins that are playing role in controlling the cell cycle progression and DNA replication events. And also it can be claimed that any change in Cyclin D1 interaction with these proteins can cause change in its associated pathway which can lead to onset of cancer.

**Table 7:** Prediction of molecular interaction of Cyclin D1 with other proteins using STRING

Predicted functional partners	Confidence scores*	Prediction for specific action
CDK4 Cyclin-dependent kinase 4	0.995	Binding, activation, catalysis, reaction, inhibition, expression with inhibition, and post-translational modification
CDK6 Cyclin-dependent kinase 6	0.880	Binding, post-translational modification, catalysis, reaction, and inhibition
CDKN1A Cyclin-dependent kinase inhibitor 1	0.993	Binding, activation, catalysis, reaction, and inhibition
IFNAR1 Interferon alpha/beta receptor 1	0.993	No direct predicted action
MCM10 Protein MCM10 homolog	0.872	No direct predicted action
CDK2 Cyclin-dependent kinase 2	0.853	Binding, activation, catalysis, reaction, and inhibition
CDKN1B Cyclin-dependent kinase inhibitor 1B	0.689	Binding, inhibition catalysis, and reaction
CDKN1C Cyclin-dependent kinase inhibitor 1C	0.814	Binding, reaction, and inhibition
CDK1 Cyclin-dependent kinase 1	0.781	Binding, activation, catalysis, reaction, and inhibition
ORC4 Origin recognition complex subunit 4	0.722	No direct predicted action

* Confidence score = how likely STRING judges an interaction to be true. 0–1, where 1 is highest confidence and ≤ 0.5 represents a false positive interaction.

#### Damaging effect of SNP on post-translational modifications sites

The effect of deleterious SNPs on having putative phosphorylation, methylation, sumoylation, and ubiquitination sites was evaluated for change in post-translation modification of Cyclin D1. GPS 5.0 has predicted the significant gain of serine phosphorylation site at R15 and A190 when mutated (R15S and A190S) which was also confirmed by Netphos 3.1. These sites on mutation R15S and A190S are predicted to cause Cyclin D1 prone to Protein Kinase G and Protein kinase phosphorylation, respectively.

In WT Cyclin D1, the GPS-MSP has predicted the R15 and R179 as methylation sites and change in amino acid (R15S and R179H/L) at these sites has predicted to cause loss of methylation function. ModPred has only predicted R179 (score 0.58) as methylation site and upon mutation the loss of methylation will take place. GPS-SUMO and UbPred has not predicted any WT SNP site as sumoylation before or after mutation. The sumoylation and ubiquitination sites were also not predicted by ModPred.

The overall impact of these missense SNPs on post-translational modification of Cyclin D1 can be stated as R15S and A190S can cause a gain of serine phosphorylation sites and loss of methylation function which can lead to cancer progression. While other SNPs has shown no effect on post-translational modification of Cyclin D1.

#### SNPs impact on Cyclin D1 domain

**Figure 5:**
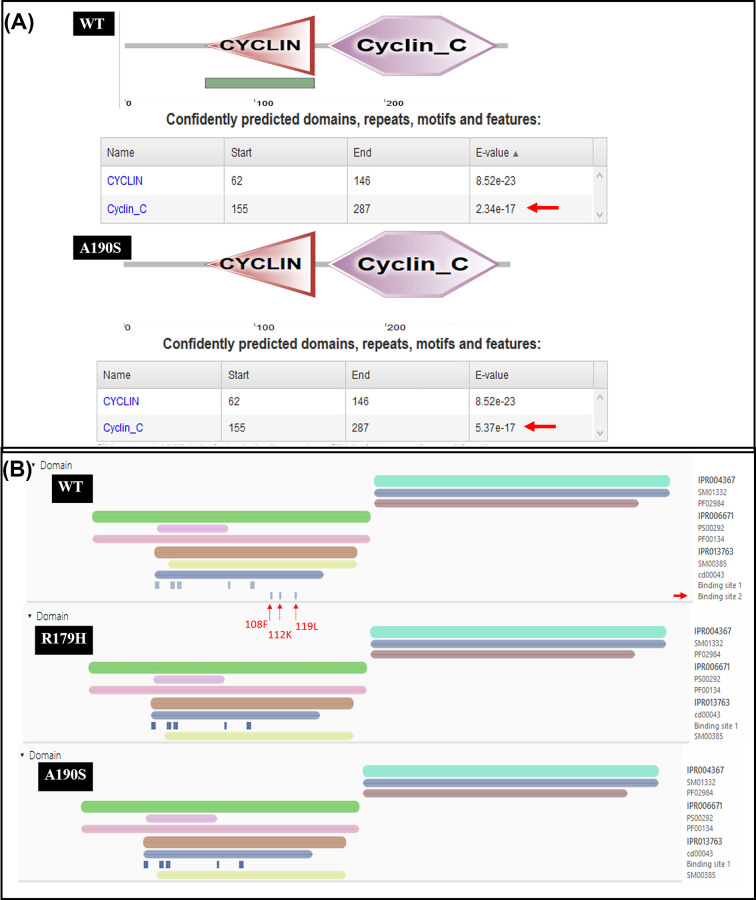
The prediction of SNPs effect on domains of Cyclin D1 using SMART (**A**) SMART domain analysis showing a change in *e*-value of Cyclin C domain due to A190S SNP. The red arrows indicating the change in *e*-value. (**B**) InterPro analysis showing the missing binding sites due to R179H and A190S SNP. The red arrows indicating the binding sites which become missing from Cyclin D1 protein due to A179S and A90S SNPs.

The WT structure of Cyclin D1 is predominantly consisted of Cyclin domain. Thus, we employed SMART analysis which recognized the Cyclin and Cyclin C domains consisting of 62-146 and 155 to 287 amino acids, respectively, in WT protein structure. These domains were found unaffected by all the four SNPs. However, a non-significant change in *e*-value was noticed due to A190S SNP. ScanProsite detected the Cyclin domain ranging 57-88 amino acid and no effect of SNP was found in this domain. InterPro has shown cyclone-like (IPR013763), Cyclin-N (IPR006671) and Cyclin-C terminal (IPR004367) domain in WT Cyclin D1. These domains were found unaffected by the A15S and D292E SNPs. However, due to R179H, R179L, and A190S SNPs the missing binding sites from Cyclin-like domain (56–131 amino acid) was predicted that is shown in Figure 5. These binding sites are located at the Cyclin box fold which is an integral part of protein binding domain functioning in cell-cycle and transcription control, present in Cyclins, TFIIB and RB. These missing binding sites are positioned at 108, 112, and 119 corresponding to the F, K and L residue respectively. So this can be interpreted that these SNPs might somehow effect the regulation of CDKs. Although these SNPs were not found to effect the domain of Cyclin D1 dominantly; however, A190S may somehow results in change in domain binding site modification.

#### SNP impact on secondary and tertiary structure of Cyclin D1

**Figure 6:**
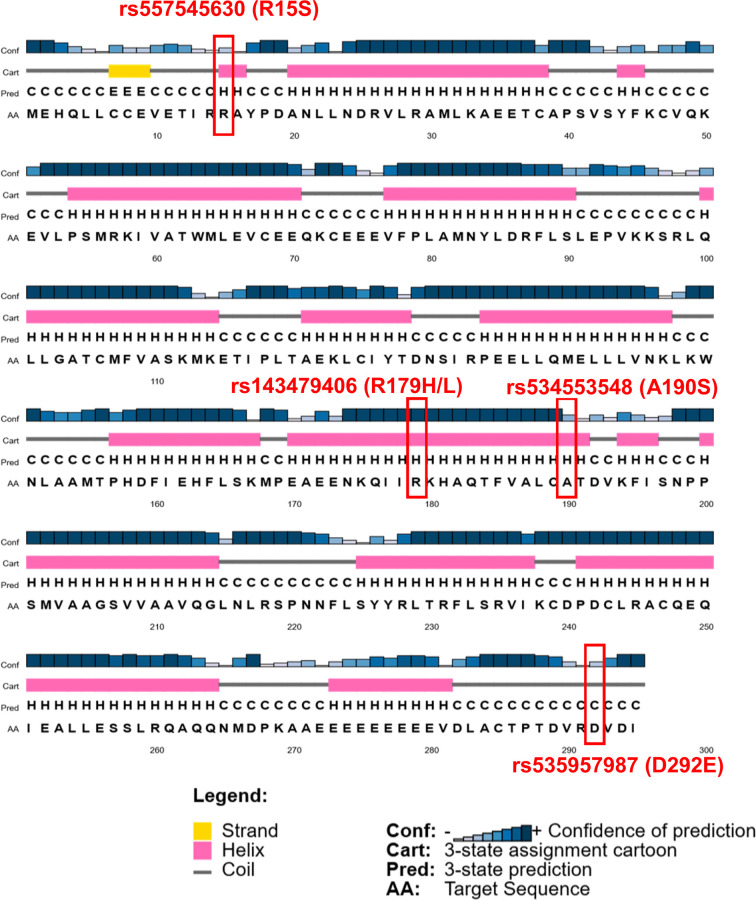
The secondary structure of Cyclin D1 predicted by the PSIPRED The red rectangles representing the position of SNP.

**Figure 7:**
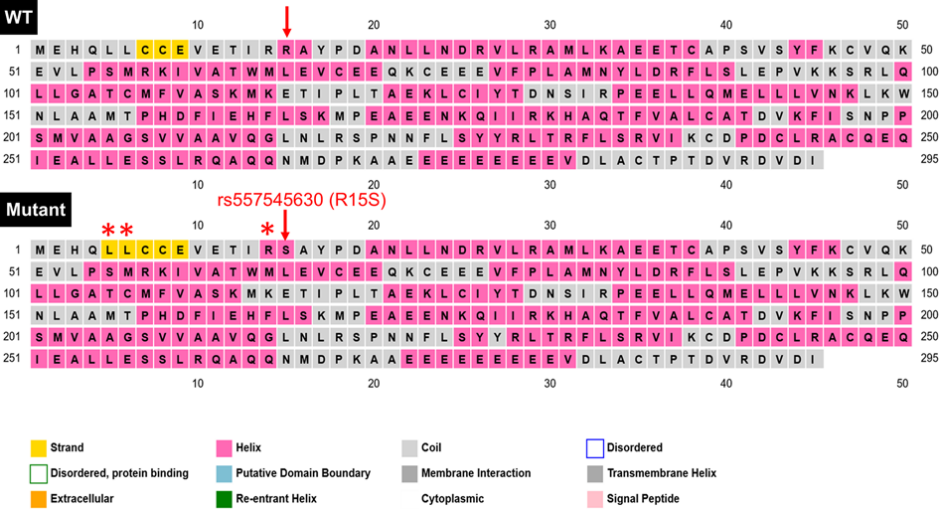
PSIPRED sequence plot showing the change in secondary stricture of Cyclin D1 due to R15S SNP Red arrows indicating the position of SNP. Asterisk (*) representing the location of change of secondary structure in mutant Cyclin D1.

**Figure 8:**
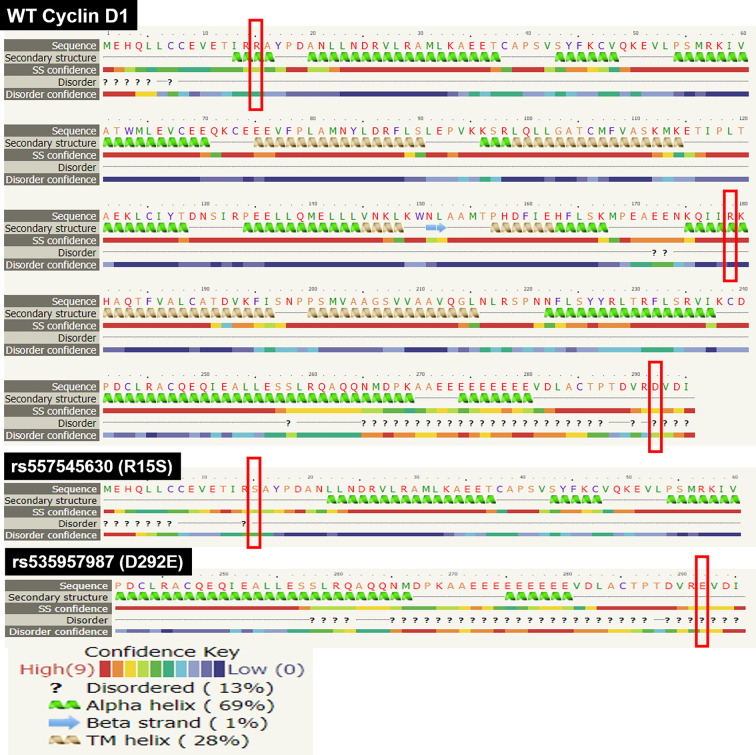
Pyrex secondary structure prediction for WT Cyclin D1 and the two SNPs (R15S and D292E) that have shown effect on secondary structure The red boxes representing the position of SNPs.

Approximately 80% of disease causing variants in amino acid sequence are found in the secondary structure of a protein [16]. Therefore, the effect of SNP on secondary structure of a protein is highly needed to understand any change in tertiary structure of that protein. In this research study, the WT secondary structure analysis by PSIPRED has predicted the helical structure of Cyclin D1 at position R15, R179 and A190, while coiled at D292 as shown in Figure 6. The alternations at these positions has not shown any change in secondary structure conformation except one SNP that is R15S which has caused a secondary structure change in nearby residues as well, that has been shown in sequence plot of PSIPRED in Figure 7. Due to this SNP, the L5 and L6 have been changed from coiled to extracellular conformation; R14 has been changed from coil to helix structure; and A16 from helix to coiled structure change has been predicted. On contradiction, the Phyre.2 analyses has predicted that R15S SNP has caused a loss of alpha helix from position I13, R14, R15 and A16, and this change is also predicted as disordered to native Cyclin D1. The D292E SNP has not caused any change in secondary structure but predicted to have a disordered effect (Figure 8). According to Missense3D prediction, A190S SNP has caused a contraction of cavity volume by 24.192 Å^3^, while R179H SNP has break a salt bridge. The WT salt bridge between NH1 atom of R179 and OE1 atom of E172 has been altered to salt bridge between ND1 atom of H179 and OE1 atom of E162 (distance: 4.595 Å) by A179H SNP. However, overall no structural damage of Cyclin D1 by these SNPs was predicted by Missense3D analysis.

**Figure 9:**
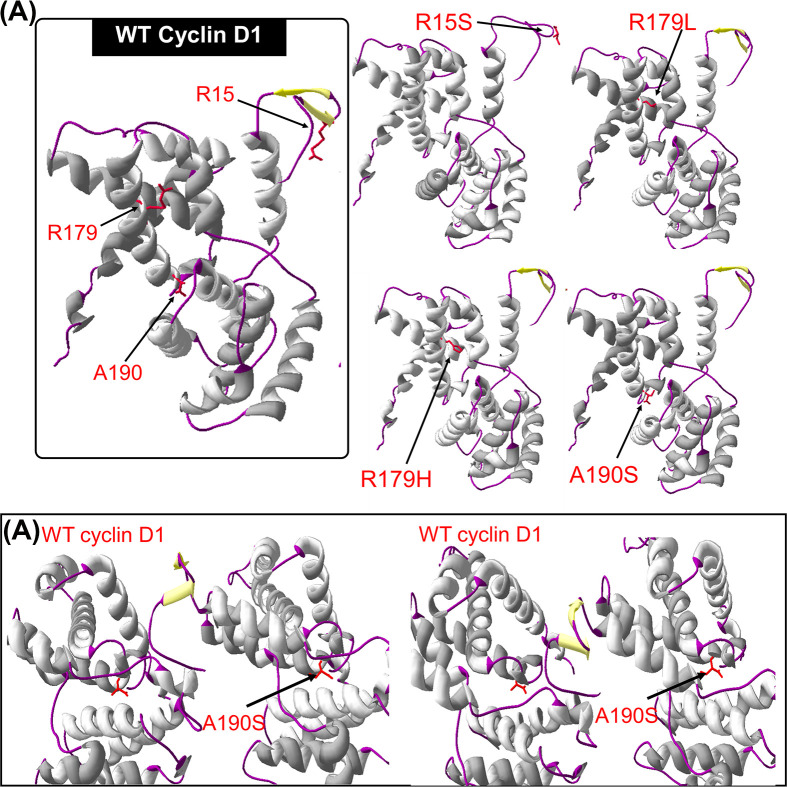
The Swiss-modelling prediction of Cyclin D1 tertiary structure (**A**) The comparison of WT Cyclin D1 structure with mutant Cyclin D1 due to SNPs. (**B**) The comparison on WT and mutant Cyclin D1 structure due to A190S from different angels, representing the contraction in cavity.

**Figure 10:**
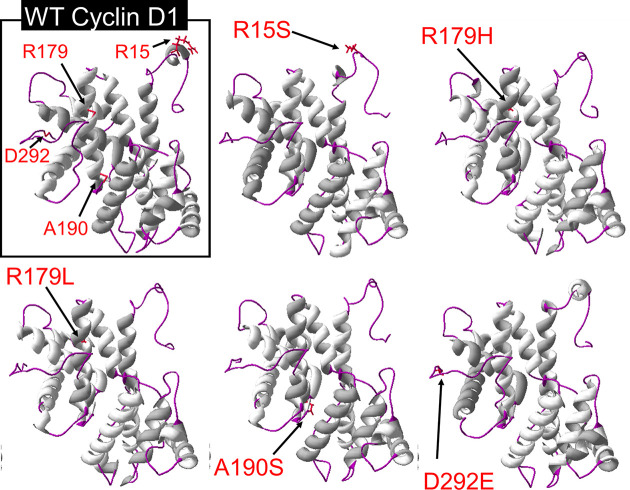
The tertiary structures of WT and mutant Cyclin D1 predicted by I-Tasser

The homology modelling was performed using Swiss-modelling and CPH models. The tertiary structure of Cyclin D1 predicted by Swiss-modelling was from 1 to 265 amino acid (Figure 9), and CPH has predicted the model from 26 to 265 amino acid (Supplementary Figure S1). The structural models of WT Cyclin D1 and mutant predicted by Swiss-modelling are shown in Figure 9. The Swiss-modelling has shown the missing beta sheets due to R15S SNP. This change in Cyclin D1 structure may cause a disruption in protein–protein interaction and other functions [17]. A slight change in protein structure can also be visualized due to A190S SNP, which has caused a contraction in cavity as shown in Figure 9B, where the structure has been visualized from two different directions. These results also confirm the prediction of Phyre.2. For further confirmation of structural predictions, I-Tasser was accessed. The secondary structure results predicted from I-Tasser were also verifying the predictions of Phyre.2. The SNP R15S has cause a loss of helix and gain of coil structure. However, the R179H/L and A190S were also predicted to cause a loss of helix structure effecting amino acid from 12 to 15. I-Tasser has also predicted that these SNPs has not caused any change in solvent accessibility of WT structure. The structural predictions from I-Tasser tool has shown a loss of helix structure around R15 position, due to R15S, R179H/L, and A190S SNPs (Figure 10).

The homology modelling and structural analysis has not predicted a very major change in structure of Cyclin D1 due to these SNPs; however, comparatively R15S and A190S may impact the Cyclin D1 conformation more. Furthermore, as predicted earlier by other *in silico* tools, four SNPs can effect stability, post-translational modification events, and evolutionary conservation of Cyclin D1.

### Frameshift mutations

Out of total four frameshift SNPs of Cyclin D1, two were found to be in MAF range (0.0001–0.5) and were selected for structural and functional impact on protein. These two SNPs are rs1565225330 (p.Q176fs) and rs1448866519 (p.L229fs).

MutationTaster has predicted these both SNPs as disease causing that has significantly changed the amino acid sequence and has led to truncated protein. Both of these mutations were also predicted to cause a change in splice site region. Q176fs has cause a gain of donor splice site AT (gDNA position 2852), while L229fs has caused a gain of accepter and donor sites at different splice site regions of Cyclin D1. L229fs was also predicted to cause an existing acceptor site stronger at gDNA position 7016. MutationTaster also predicts the regulatory feature effected by SNPs. These regulatory feature determined a protein to bind to specific DNA sequences and control to switch on genes under any particular conditions. These frameshift SNPs were predicted to possibly affect the histone modification and RNA polymerase feature of Cyclin D1. Q176fs has predicted to cause a change in histone 3 lysine 9 acetylation, histone 3 lysine 4 tri-methylation, histone 3 lysine 36 tri-methylation, histone 3 lysine 4 di-methylation, histone 3 lysine 27 acetylation, histone 3 lysine 79 di-methylation, and RNA polymerase II binding, while L229fs has cause destruction of histone 3 lysine 36 tri-methylation and RNA polymerase II binding. Previously, it has been reported that histone acetylation and histone methylation may inhibit human D1 transcription [18]; therefore, this change in Cyclin D1 regulatory features is predicted to impact it functional activities. MutationTaster uses values from phastCons and phyloP to determine the grade of conservation of a given nucleotide. According to its evolutionary conservation prediction, both the SNPs have caused an alternation at very highly conserved nucleotide.

**Figure 11:**
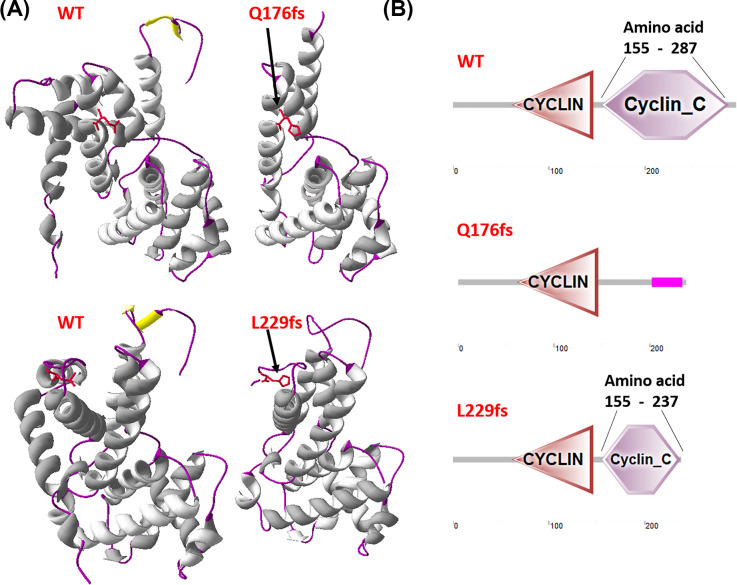
Frameshift SNPs effect on Cyclin D1 (**A**) Prediction of tertiary structure using Swiss-modelling. (**B**) Prediction of domain changes using SMART.

The tertiary structure of mutant protein was modelled to predicted the structural impact of these two frameshift SNPs on Cyclin D1 using Swiss-modelling and change in domain was predicted by SMART. Figure 11 shows that frameshift mutation has led to formation of the truncated protein and has also lead to loss or formation of damaged Cyclin_C domain due to frameshift SNPs. The drastic change in structure of Cyclin D1 due to these SNPs will cause a loss of Cyclin D1 native function. Q176fs has caused a loss of Cyclin C domain and formed a low compositional complexity region which do not have ability to control the progression of cells through the cell cycle by activating CDK enzymes. On the other hand, L229fs has led to formation of shortened Cyclin C domain which may cause a malfunctioning of Cyclin D1.

**Figure 12:**
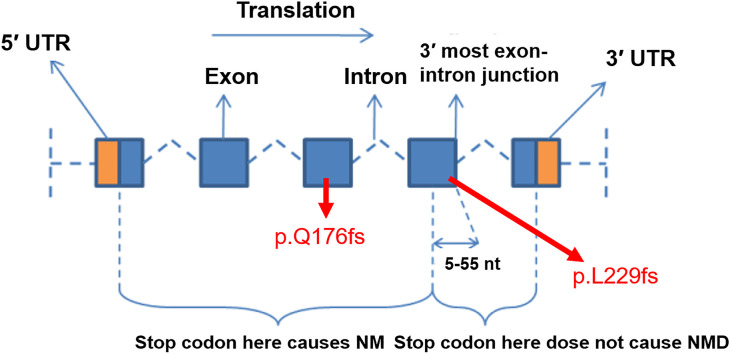
The possible location of occurrence of NMD on Cyclin D1, predicted by SIFT tool analysis The frameshift SNPs is also positioned on Cyclin D1.

Using the SIFT analysis the possibility of damaging effect of frameshift mutation and occurrence of NMD was verified. The outcome from SIFT predicted that both of the SNPs has damaging effect. It has been reported that NMD do not occur when the premature termination codon is in the last exon or it is in the last 50 nucleotides in the second to last exon [19] as shown in Figure 12. Figure 12 is displaying the five exons of the Cyclin D1 and location of possible occurrence of NMD. The position of frameshift SNPs is also mentioned at exon 2 3 and exon 4 of Cyclin D1. The mRNA transcripts that contains the stop codons are eliminated or degraded due to the initiation of NMD which results in the limit of translation of abnormal protein. Q179fs, therefore, may cause NMD and reduce formation of truncated protein.

The structural and functional prediction of frameshift SNPs describes that these two SNPs are forming a truncated Cyclin D1 which is possibly effecting its native function.

#### UTR SNP effect on Cyclin D1

The UTR SNPs were selected on basis of MAF range. The 116 SNPs of 3′ and 11 SNPs of 5′ UTR SNPs were analyzed using Variowatch, PolymiRTS, and RegulomDB server. The RegulomDB analysis shows that 3′ and 5′ UTR SNPs have not any strong impact to change the regulatory function of *CCND1*. The results also showed that 53/116 SNPs of 3′ UTR and 1/11 SNP of 5′ UTR are less likely to be functional and effect biding (categories 2a, 2b, and 3a) and 63/116 SNPs of 3′ UTR and 10/11 SNPs of 5′ UTR has minimal functional evidence (categories 4, 5, and 6). According to the Variowatch and PolymiRTS database prediction, the 3′ and 5′ UTR SNPs have no impact on mRNA stability, interaction (protein–mRNA and miRNA–mRNA) or any influence in *CCND1* expression level. The overall prediction analysis shows that *CCND1* UTR SNPs have no major impact on translation.

#### Clinical correlation between *CCND1* SNP and patients with breast cancer

*CCND1* SNPs were investigated at NCBI to sort out the clinical significant SNPs. Only one SNPs (rs9344) have been reported to have clinical significance. rs9344 is a synonymous SNP and has been associated as a risk factor SNP for different types of cancer [20–25] in different population including breast cancer [22,26]. In Chinese population, this SNP did not show to have a major association with breast cancer. But it has been evaluated that this SNP has an effect of estrogen on breast cancer growth and after diagnosis it predict the survival of patients with breast cancer [27]. Mining the data in GDC data portal, it was found that only one SNP (rs755986542, R260C) was entitled in TCGA data and has shown an in-significant correlation with breast cancer clinically. In a project cases tested for Simple Somatic Mutation (SSM), out of 986 clinical breast cancer cases only 1 case (0.10%) was found as carrier of this SNP, suffering from ductal and lobular neoplasms of stage I.

There are thousands of GWAS studies that associate variants with traits. The most accurate and significant findings (*P*-value <10^−5^) are accomplished by the summary of statistics provided by the association study [28]. In this research study, the comprehensive search was performed to collect all the SNPs of *CCND1* that are associated with breast cancer by GWAS. There are total five *CCND1* SNPs (rs614367, rs75915166, rs554219, rs78540526, and rs34507830) that have shown an association with breast carcinoma in five different GWAS studies as shown in Table 8. It was found that these SNPs belongs to noncoding region, regulatory region, and transcription binding site region. According to the GWAS, it was also found that among different cancers *CCND1* has highest association with breast cancer.

**Table 8:** Association of *CCND1* SNPs reported by GWAS

SNPs	Consequence	*P*-values*	Study accession	Studies
**rs614367** **g.69513996C>T**	Intergenic variant	3 × 10^−15^	GCST000678	(Turnbull et al. 2010)
		2 × 10^−63^	GCST001937	(Ahsan et al. 2014)
		1 × 10^−8^	GCST002346	(Michailidou et al. 2013)
**rs75915166** **g.69564393C>A**	Tf-binding site variant	4 × 10^−95^	GCST004988	(Michailidou et al. 2015)
		1 × 10^−57^	GCST004950	(Michailidou et al. 2017)
**rs554219** **g.69516874C>A**	Regulatory region variant	6 × 10^−47^	GCST004988	(Michailidou et al. 2015)
		2 × 10^−81^	GCST004950	(Michailidou et al. 2017)
**rs78540526** **g.69516650C>T**	Regulatory region variant	2 × 10^−131^	GCST004988	(Michailidou et al. 2015)
		2 × 10^−86^	GCST004950	(Michailidou et al. 2017)
		2 × 10^−62^	GCST007236	(Michailidou et al. 2015)
**rs34507830** **g.69646918C>T**	Intron variant	7 × 10^−31^	GCST004988	(Michailidou et al. 2017)

* Significant *P* value <10^−5^

**Figure 13:**
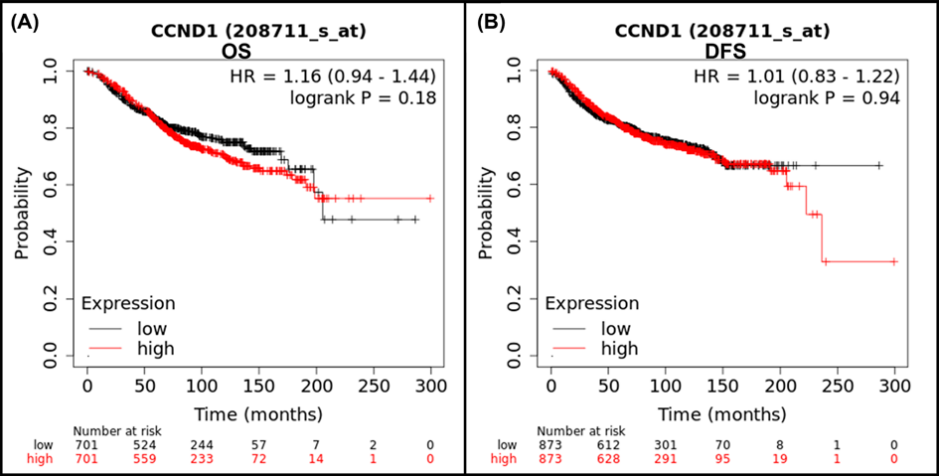
Kaplan–Meier plotter displaying the expression of Cyclin D1 and survival time of patients with breast cancer (**A**) Overall survival (OS) of patients with breast cancer (*n*=1402). (**B**) Disease-free survival (DFS) of patients with breast cancer (*n*=1746). On the basis of selected parameters, the analysis was run on ‘*n*’ number of patients with available clinical data; HR, hazard ratio.

In the present study, the impact of *CCND1* on survival of patients with breast cancer was investigated using Kaplan–Meier plotter. In Figure 13, the red lines indicate the survival time of patients with breast cancer with high *CCND1* expression levels, and black lines indicate the survival time of patients with breast cancer with low *CCND1* expression levels. Low expression of *CCND1* (0.1761) was found to be correlated with better overall survival (OS) for patients with breast cancer (*n*=1402). A significant difference was observed between OS and disease-free survival (DFS) of patients with breast cancer (*n*=1746), which indicates that the patients with *CCND1* alternations has improved prognosis as compared with those without *CCND1* alternations. However, a strong difference in curve is noted between low and high expression level, which shows that the high expression of *CCND1* is found to be associated with high number of patients at risk which gives a less survival rate for patients with breast cancer.

**Figure 14:**
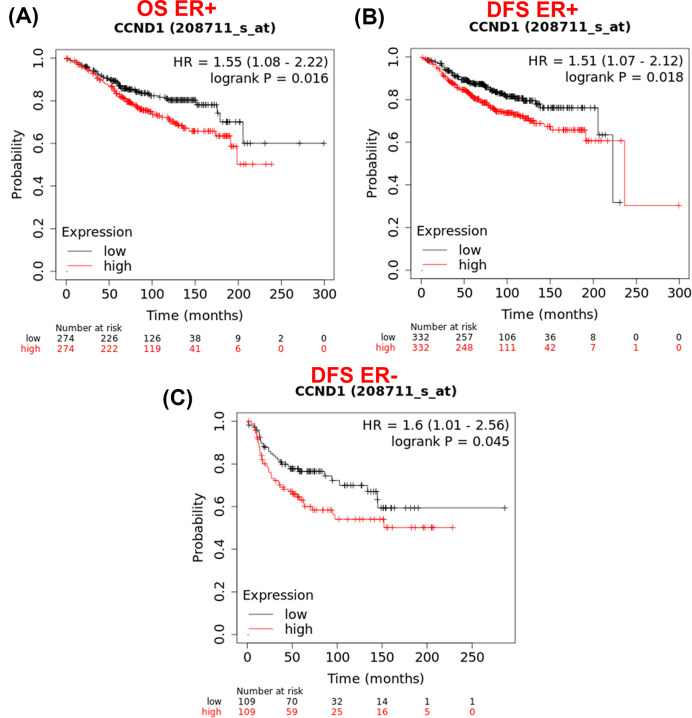
The graphs displaying the positive association of Cyclin D1 expression in patients with breast cancer with different clinical parameters evaluated by Kaplan–Meier plotter The OS of patients with breast cancer association with (**A**) ER+ (*n*=548), and DFS of patients with breast cancer association with (**B**) ER+ (*n*=664) and (**C**) ER- (*n*=218). On the basis of selected parameters, the analysis was run on ‘*n*’ number of patients with available clinical data; DFS, disease-free survival; OS, overall survival.

To further investigate the association of Cyclin D1 expression in breast cancer patients with different clinical parameters, HER2- (*P*=0.083), ER- (*P*=0.016), and ER+ (*P*=0.067) were found significant associated with OS and ER+ (*P*=0.018) and ER- (*P*=0.045) were significant with DFM in breast cancer (Figure 14). While there was no statistical evidence which reveals the association of PR and HER2 with DFM with expression of Cyclin D1 in patients with breast cancer (Supplementary Figure S2). The prognostic factors association with *CCND1* expression is also shown by cox multivariate analysis given in Table 9.

**Table 9:** Cox multivariate analysis of prognostic factor association with *CCND1* expression

	OS			DFS		
	HR	95% CI	Log rank*	HR	95% CI	Log rank*
**ER**+	1.55	1.08–2.22	0.016	1.51	1.07–2.12	0.018
**ER-**	1.54	0.97–2.44	0.067	1.6	1.01–2.56	0.045
**HER2**+	1.28	0.63–2.62	0.49	0.89	0.47–1.68	0.72
**HER2-**	2.27	0.88–5.89	0.083	1.32	0.56–3.13	053
**PR**+	1.4	0.37–5.29	0.62	1.07	0.47–2.42	0.88
**PR-**	1.22	0.48–3.08	0.68	1.1	0.62–1.97	0.74
**Grade 3**	1.25	0.9–1.74	0.18	1.25	0.88–1.77	0.21
**Lymph node status**	1.16	0.78–1.72	0.46	0.81	0.55–1.19	0.27

* Significant *P*<0.05

Abbreviations: DFS, disease-free survival; HR, hazard ratio; OS, overall survival.

The variants with unknown clinical significance are classified by the American College of Medical Genetics as Variants of Uncertain Significance (VUS). The *in silico* tools, like SIFT, PolyPhen-2 and FATHMM, have been used previously to predict the impact of VUS on pathogenicity of its mutant protein, while their use to predict the clinical significance of a particular SNP or mutation is still unclear [29]. In this research study, on basis of analysis from these tools, out of 169 missense SNPs of *CCND1* the 77 have been predicted as highly deleterious SNPs and have been associated with a diseased phenotype (Supplementary Table S2). To further significantly validate the SNP with its clinical importance, an extensive *in vitro* and *in vivo* research study is needed.

## Discussion

Breast cancer is worldwide emerging disorder of women with approximately 2.1 million new cases are diagnosed and almost 0.6 million deaths in each year [30]. The overall survival rate of patients with breast cancer has reached up to 90%,;however, metastatic or advanced breast cancer survival rate is still 25% [31]. Recent therapeutic approach which includes endocrine therapy and targeted therapy is productive in prognosis of breast cancer. Furthermore, Cyclin-dependent kinase such as CDK4 and CDK6 inhibitors also showed clinical benefits [32]. In the present study, we have collected *CCND1* SNPs data from the NCBI genome workbench and applied *in silico* analysis on coding nonsynonymous SNPs, splice site SNPs and 5′ and 3′ UTR SNP to predict their pathogenic impact on protein structure and function in relation with breast cancer.

Our results displayed that four SNPs reside in highly conserved region of Cyclin D1. However, only R15S and A190S have displayed a significant diseased phenotype and an altered molecular mechanism predicted by MutPred, FATHMM, SNPeffect, SNAP2, and PredictSNP. Further analysis indicated that A190S, R179L, and R15S may also cause a decrease in stability of Cyclin D1 anticipated by I-Mutant, HOPE, and SNPeffect. Previous study on the mutation in splicing region showed that the improper exon and intron recognition occurred by splicing machinery which results in an aberrant transcript. These variants may also interrupt the existing splice sites, create new ones and also it can impact splicing enhancers and silencers binding. Therefore, the variants in splice site accounted for most of the diseases [15]. Generally, the variants residing within 10 nucleotide position in intronic splice site region account more in creating defective splice site [15]. The two most occurring splice transcripts of *CCND1* are Cyclin D1a and Cyclin D1b. Cyclin D1a contains all the 5 exons but Cyclin D1b formed by intron 4 inclusion and exon 5 skipping which is modulated by Serine and arginine rich splicing factor 1 (SRSF1) for up-regulation of Cyclin D1b in breast cancer. The Cyclin D1b is a product of alternate splicing due to silent polymorphism G/A870, in which the A allele was assumed to reduce the efficacy of the splice donor site [33]. In our study, 23 SNPs out of total 2506 intronic SNPs were found within 10 nucleotide position at 5′ and 3′ region of intron. The SNP declared as damaging by three or more *in silico* tools out of five (HSF, Sroogle, SliceView, NetGene2, and FSplice) is considered as highly pathogenic and may effects the splicing region. The *in silico* analysis of these tools has predicted that the SNP can affect the donor or acceptor site of splice region of *CCND1*.

In molecular interaction analysis, Cyclin D1 is predicted to be directly involve in binding, reaction, catalysis, and inhibition. It is also take part in post-translational modification in association with CDK4/6. The CDK plays a vital role in controlling the G1/2-S transition in cell cycle, promotes the E2F transcriptional program, and the initiation of DNA synthesis. These are also found to be involved in the assembly, stability, and modulation of DC activation. Out of these proteins, the CDK4 is a regulatory component of the DC complex which is a major integrator of various mitogenic and antimitogenic signals, plays a vital role in cancer [34,35]. We performed the STRING analysis which demonstrated that Cyclin D1 remain in strong association with the proteins that play vital role in cell cycle progression and DNA replication events. Thus, it can be suggested that any change in Cyclin D1 interaction with these proteins may change its associated pathway which can lead to onset of cancer. The WT structure of Cyclin D1 is predominantly consisted of Cyclin domain. The Cyclin domain is generally present in Cyclins proteins, and also transcription factor IIB (TFIIB) and retinoblastoma (RB). These functioning in cell-cycle, transcription control, and cancer progression [36]. In order to understand the role of Cyclin domain of *CCND1*, SMART software was used and analysis revealed that Cyclin and Cyclin C domains consisting of 62–146 and 155–287 amino acids, respectively, in WT protein structure. These domains remain unaffected by our SNP analysis; however, a nonsignificant change in *e*-value was noted. Furthermore, ScanProsite spotted the Cyclin domain range from 57 to 88 amino acid while no effect of SNP was observed in this domain. InterPro has shown cyclone-like (IPR013763), Cyclin-N (IPR006671), and Cyclin-C terminal (IPR004367) domain in WT Cyclin D1. These domains were found unaffected by the A15S and D292E SNPs. What is more, effect of damaging SNP was predicted on secondary structure Cyclin D1 and we observed contraction of cavity volume while homology modelling, and structural analysis has not predicted a very major change in structure of Cyclin D1. Next, we studied survival of patients with breast cancer and prognosis prediction using Kaplan–Meier plotter. We found low expression of *CCND1* (0.1761) was found to be correlated with better OS for patients with breast cancer (*n*=1402). Further analysis indicated that there is a significant difference between OS and DFS of patients with breast cancer (*n*=1746), which indicates that the patients with *CCND1* alternations has improved prognosis as compared with those without *CCND1* alternations.

Altogether, out of 3747 SNPs of *CCND1*, only one splice site SNP rs752676953 (c.1988+5G<A) and two frameshift SNPs, rs1565225330 (p.Q176fs) and rs1448866519 (p.L229fs), have predicted to be strongly effect the splice site. Two missense SNPs rs557545630 (R15S) and rs534553548 (A190S) have diseased phenotype which may also affect the post-translational modification of Cyclin D1. A190S was predicted to cause a major change in stability of the Cyclin D1 and may somehow bring changes in domain binding site. No major change in structure of Cyclin D1 by these missense SNPs was predicted; however, two frameshift SNPs have resulted truncated Cyclin D1 protein structure. These changes might affect the Cyclin D1 interaction with other proteins and can disrupt its function as well. None of these SNPs were previously related with breast cancer. However, GWAS study has reported 5 SNPs (rs614367, rs75915166, rs554219, rs78540526, and rs34507830) to be significantly associated with breast cancer. The Kaplan–Meier plotter has explained that high expression of *CCND1* is associated with less survival rate of breast cancer patients. Our study suggests that c.1988+5G<A, R15S, R179L, and A190S SNPs could directly or indirectly destabilize the Cyclin D1. If promise of the computational analysis of SNPs is to be realised, this information can be integrated into *in vivo* and *in vitro* analysis to further validate and implement these SNPs in treatment or prognosis of breast cancer.

## Supplementary Material

Supplementary Figures S1-S2 and Tables S1-S3

## Abbreviations

DFM: disease-free survival
HR: hazard ratio
OS: overall survival
SNP: single-nucleotide polymorphism
SS: splice site
