# Supplementary material for: Computational analysis of Cyclin D1 gene SNPs and association with breast cancer
Source: Biosci Rep. 2021 Jan 29;41(1):BSR20202269. doi: 10.1042/BSR20202269 (PMC7846961; doi:10.1042/BSR20202269)
Supplement: Supplementary Figures S1-S2 and Tables S1-S3 [file BSR-2020-2269_supp.pdf]

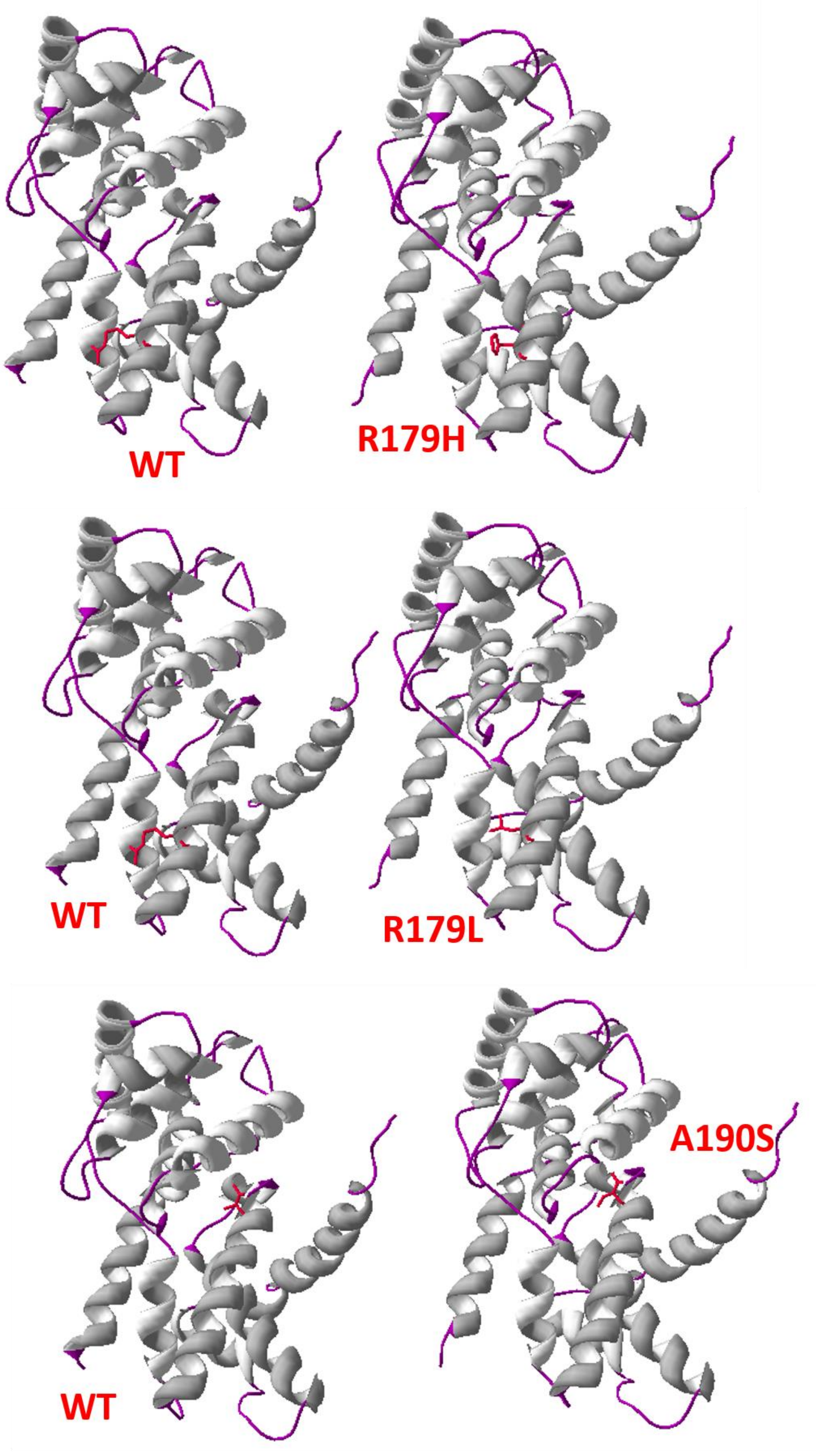
WT
R179H
WT
R179L
A190S
WT

Supplementary Figure 1. The CPH model of WT cyclin D1 structure, and mutant cyclin D1 structures due to R179H, R17L and A190S SNPs

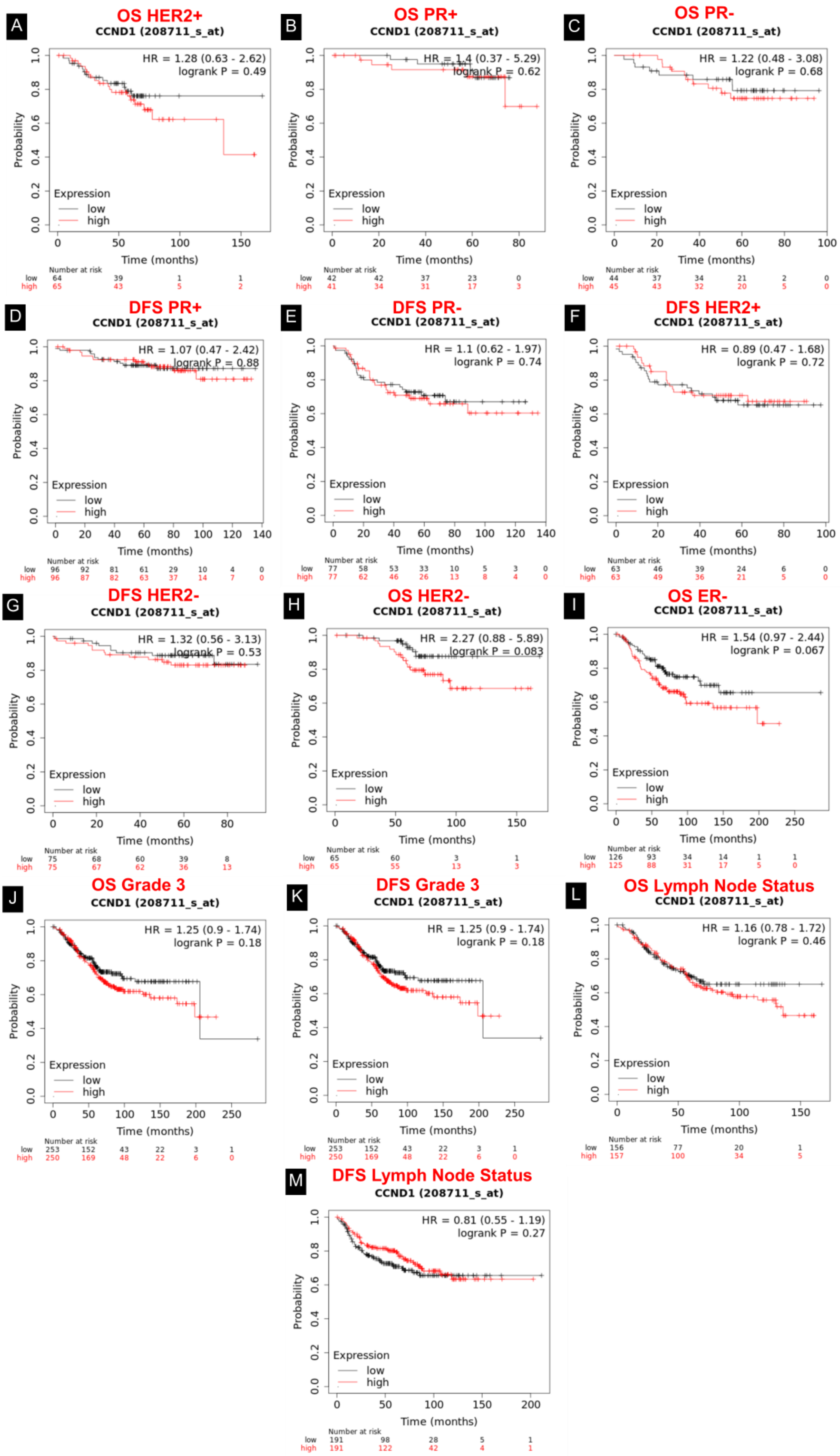
A
OS HER2+
CCND1 (208711_s_at)
HR = 1.28 (0.63 - 2.62)
logrank P = 0.49
B
OS PR+
CCND1 (208711_s_at)
HR = 1.4 (0.37 - 5.29)
logrank P = 0.62
C
OS PR-
CCND1 (208711_s_at)
HR = 1.22 (0.48 - 3.08)
logrank P = 0.68
D
DFS PR+
CCND1 (208711_s_at)
HR = 1.07 (0.47 - 2.42)
logrank P = 0.88
E
DFS PR-
CCND1 (208711_s_at)
HR = 1.1 (0.62 - 1.97)
logrank P = 0.74
F
DFS HER2+
CCND1 (208711_s_at)
HR = 0.89 (0.47 - 1.68)
logrank P = 0.72
G
DFS HER2-
CCND1 (208711_s_at)
HR = 1.32 (0.56 - 3.13)
logrank P = 0.53
H
OS HER2-
CCND1 (208711_s_at)
HR = 2.27 (0.88 - 5.89)
logrank P = 0.083
I
OS ER-
CCND1 (208711_s_at)
HR = 1.54 (0.97 - 2.44)
logrank P = 0.067
J
OS Grade 3
CCND1 (208711_s_at)
HR = 1.25 (0.9 - 1.74)
logrank P = 0.18
K
DFS Grade 3
CCND1 (208711_s_at)
HR = 1.25 (0.9 - 1.74)
logrank P = 0.18
L
OS Lymph Node Status
CCND1 (208711_s_at)
HR = 1.16 (0.78 - 1.72)
logrank P = 0.46
M
DFS Lymph Node Status
CCND1 (208711_s_at)
HR = 0.81 (0.55 - 1.19)
logrank P = 0.27
Probability
Time (months)
Expression
low
high
Number at risk

Supplementary Figure 2. The association of cyclin D1 with different clinical parameters in breast cancer patients. OS association with **A)** HER+ (n= 129), **B)** PR+ (n= 83) and **C)** PR- (n= 89). DFM association of breast cancer patients with **D)** PR+ (n= 192), **E)** PR- (n= 154), **F)** HER+ (n= 126), and **G)** HER- (n= 150)

Supplementary Table 1. *In silico* analysis of intronic splice region *CCND1* SNPs.

**No.**	**RS ID**	**SNP**	**HSF [a] HSF matrix score (0-100)**				**SpliceView [b] Score (0-100)**		**Sroogle [c]**			**NetGene2 [d] confidence score (0-1)**		**FSplice [e] (Weight index)**		**Prediction of possible impact [f]**
			**WT**	**Mu**	**Variation %**	**Interpretation**	**WT**	**Mu**	**Element**	**WT**	**Mu**	**WT**	**Mu**	**WT**	**Mu**	
1	rs374405138	g.5411C>T, c.198+4C>T	44.25	71.08	+60.63	Potential alteration of splicing. New Donor Site	84 DS	84 DS	5´ SS	-3.80 (Delta-G) 6.79 (Max entropy)	-3.90 (Delta-G) 4.48 (Max entropy)	0.70 DS	0.61 DS	7.36	6.66	No Effect
2	rs752676953	g.5412G>A, c.198+5G>A	86.34	74.18	-14.08	Probably effecting splicing	84 DS	78 DS	5´ SS	-3.80 (Delta-G) 6.79 (Max entropy)	-3.80 (Delta-G) 2.02(Max entropy)	0.70 DS	Not generated	W= 7.36	Not generated	Probably effecting
3	rs1456525574	g.5413G>A, c.198+6G>A	86.34	85.94	-0.46	Probably no impact on splicing	84 DS	83 DS	5´ SS	-3.80 (Delta-G) 6.79 (Max entropy)	-3.80 (Delta-G) 5.51 (Max entropy)	0.70 DS	0.71 DS	7.36	W=7.92	No Effect
4	rs1343339113	g.5415G>A, c.198+8G>A	No difference between mutant and reference sequence			Probably no impact on splicing	Not predicted	Not predicted	No difference between mutant and reference sequence			0.70 DS	0.52 DS	7.36	7.36	No Effect
5	rs1420743674	g.6919C>T, c.199-8C>T	82.96	82.34	-0.75	Probably no impact on splicing	84 AS	84 AS	3´ SS	9.86 (Max entropy) 80.21 (PSSM)	9.54 (Max entropy) 80.80 (PSSM)	0.96	0.96	4.03	4.03	No Effect
6	rs758963834	g.6922C>T, c.199-5C>T	82.96 AS	83.85 AS	+1.07	Probably no impact on splicing	84 AS	84 AS	3´ SS	9.86 (Max entropy) 80.21 (PSSM)	9.51 (Max entropy) 81.22 (PSSM)	0.96	0.96	4.03	4.03	No Effect
7	rs1339178943	g.6923G>A, c.199-4G>A	82.96 AS	83.03 AS	+0.08	Probably no impact on	84 AS	84 AS	3´ SS	9.86 (Max	10.31 (Max entropy)	0.96 AS	0.97 AS (H)	4.03	4.53	No Effect

						splicing				entropy) 80.21 (PSSM)	80.45 (P SSM)					
8	rs1424359226	g.7147C>G, c.414+5C>G	81.06 DS	93.08 DS	+14.83	Probably no impact on splicing	81 DS	82 DS	5´ SS	-3.60 (Delta-G) 4.51 (Max entropy)	-5.90 (Delta-G) 8.69 (Max entropy)	0.70 DS	0.99 DS (H)	6.24	12.82	No Effect
9	rs1484194988	g.7151delT, c.414+9delT	No difference between mutant and reference sequence			Probably no impact on splicing	Not predicted	Not predicted	No difference between mutant and reference sequence			0.70 DS	0.39 DS	6.24	6.24	No Effect
10	rs762325000	g.7720G>A, c.415-8G>A	85.61 AS	85.54 AS	-0.08	Probably no impact on splicing	83 AS	83 AS	3´ SS	7.44 (Max entropy) 81.57 (PSSM)	6.22 (Max entropy) 81.61 (PSSM)	0.34 AS	0.20	8.20	6.83	No Effect
11	rs1240440953	g.7723C>T, c.415-5C>T	85.61 AS	86.50 AS	+1.04	Probably no impact on splicing	83 AS	84 AS	3´ SS	7.44 (Max entropy) 81.57 (PSSM)	8.01 (Max entropy) 82.58 (PSSM)	0.56	0.56	8.20	9.57	No effect
12	rs377200375	g. 7894C>T, c.574+7C>T	56.84 DS	83.68 DS	+47.22	Potential alteration of splicing. New Donor Site	Not predicted	Not predicted	No difference between mutant and reference sequence			0.95 DS (H)	0.91 DS (H) New donor site	11.00	11.00	No effect
13	rs931931114	g.7896delG, c.574+9delG	76.89 AS	72.73 AS	-5.41	Probably no impact on splicing	Not predicted	Not predicted	No difference between mutant and reference sequence			0.95 DS (H)	0.95 DS (H)	11.00	11.00	No effect
14	rs750369077	g.7897G>T, c.574+10G>T	76.89 AS	72.59 AS	-5.59	Probably no impact on splicing	Not predicted	Not predicted	No difference between mutant and reference sequence			0.95 DS (H)	0.95 DS (H)	11.00	11.00	No effect
15	rs201881393	g.11882T>C, c.575-8T>C	89.65 AS	90.26 AS	+0.68	Probably no impact on splicing	92 AS	92AS	3´ SS	10.04 (Max entropy) 88.71 (PSSM)	9.82 (Max entropy) 88.12 (PSSM)	1.00 AS (H)	1.00 AS (H)	13.18	12.30	No effect
16	rs1443089370	g.11883G>A, c.575-7G>A	89.65 AS	89.69 AS	+0.04	Probably no impact on	92 AS	92 AS	3´ SS	10.04 (Max	9.68 (Max entropy)	1.00 AS	1.00 AS (H)	13.18	12.93	No effect

						splicing				entropy) 88.71 (PSSM)	89.06 (PSSM)	(H)				
17	rs776761881	g.11885C>T, c.575-5C>T	89.65 AS	90.54 AS	+0.99	Probably no impact on splicing	92 AS	92 AS	3´ SS	10.04 (Max entropy) 88.71 (PSSM)	10.79 (Max entropy) 89.72 (PSSM)	1.00 AS (H)	1.00 AS (H)	13.18	13.30	No effect
18	rs1398886316	g.12045G>A, c.723+7G>A	84.35 DS	85.51 DS	+1.38	Probably no impact on splicing	Not predicted	Not predicted	No difference between mutant and reference sequence			1.00 DS (H)	1.00 DS (H)	16.18	16.18<br><br>6.94 (new DS)	No effect
19	rs1389923822	g.12047G>A, c.723+9G>A	84.35 DS	72.18 DS	-14.43	Probably no impact on splicing	Not predicted	Not predicted	No difference between mutant and reference sequence			1.00 DS (H)	1.00 DS (H)	16.18	16.18<br><br>7.22 (new DS)	No effect
20	rs371455093	g.12048G>C, c.723+10G>C	84.35 DS	84.11 DS	-0.28	Probably no impact on splicing	Not predicted	Not predicted	No difference between mutant and reference sequence			1.00 DS (H)	1.00 DS (H)	16.18	16.18<br><br>5.26 (new DS)	No effect
21	rs753060017	g.15004T>C, c.724-10T>C	93.38 AS	92.67 AS	-0.76	Probably no impact on splicing	Not predicted	Not predicted	3´ SS	10.71 (Max entropy) 88.03 (PSSM)	10.63 (Max entropy) 87.02 (PSSM)	1.00 AS (H)	1.00 AS (H)	13.60 AS	12.97	No effect
22	rs571153521	g.15008T>C, c.724-6T>C	93.38 AS	94.03 AS	+0.7	Probably no impact on splicing	93 AS	93 AS	3´ SS	10.71 (Max entropy) 88.03 (PSSM)	10.98 (Max entropy) 87.93 (PSSM)	1.00 AS (H)	1.00 AS (H)	13.60 AS	10.60	No effect
23	rs764630402	g.15010T>G, c.724-4T>G	93.38 AS	93.41 AS	+0.03	Probably no impact on splicing	93 AS	93 AS	3´ SS	10.71 (Max entropy) 88.03 (PSSM)	11.20 (Max entropy) 87.51 (PSSM)	1.00 AS (H)	1.00 AS (H)	13.60 AS	10.60	No effect

Supplementary Table 2. *In silico* analysis of *CCND1* reported missense SNPs.

	RS ID	Missense substitutions	PolyPhen-2 [a]		SIFT [b]		PROVEAN [c]		SNP & GO [d]		PANTHER [e]		Prediction of possible impact [f]
			scores	Prediction	Scores (0-1)	Prediction	Score	Prediction	Probability	Prediction	Probability	Prediction	
1	rs572037183	NM_053056.2:c.4G>A, NP_444284.1:p.Glu2Gln	0.145	Benign	0.15	Tolerant	-0.84	Neutral	0.581	Disease	NA	Unclassified	Benign
		NM_053056.2:c.4G>C, NP_444284.1:p.Glu2Lys	0.023	Benign	0.13	Tolerant	-0.96	Neutral	0.803	Disease	NA	Unclassified	Benign
2	rs545664320	NM_053056.2:c.8A>G, NP_444284.1:p.His3Arg	0.244	Benign	0.37	Tolerant	-1.3	Neutral	0.665	Disease	NA	Unclassified	Benign
3	rs1356268873	NM_053056.2:c.12G>T, NP_444284.1:p.Gln4His	0.904	Probably Damaging	0.02	Damaging	-1.49	Neutral	0.431	Neutral	NA	Unclassified	Benign
4	rs993495966	NM_053056.2:c.19T>A, NP_444284.1:p.Cys7Ser	0.999	Probably Damaging	0.01	Damaging	-6.75	Deleterious	0.978	Disease	NA	Unclassified	HighlyDamaging
5	rs1486252402	NM_053056.2:c.20G>C, NP_444284.1:p.Cys7Ser	0.999	Probably Damaging	0.01	Damaging	-6.75	Deleterious	0.978	Disease	NA	Unclassified	Highly Damaging
6	rs773884084	NM_053056.2:c.22T>G, NP_444284.1:p.Cys8Gly	0.021	Benign	0.06	Tolerant	-6.46	Deleterious	0.978	Disease	0.421	Neutral	Benign
7	rs1340132260	NM_053056.2:c.23G>A, NP_444284.1:p.Cys8Tyr	0.919	Probably Damaging	0.19	Tolerant	-5.69	Deleterious	0.958	Disease	0.289	Neutral	Highly Damaging
8	rs761266790												
9	rs1372181670	NM_053056.2:c.40C>A, NP_444284.1:p.Arg14Ser	0.411	Benign	0.74	Tolerant	-2.13	Neutral	0.879	Disease	0.282	Neutral	Benign
10	rs557545630	NM_053056.2:c.43C>A, NP_444284.1:p.Arg15Ser	0.994	Probably Damaging	0.01	Damaging	-3.8	Deleterious	0.957	Disease	0.361	Neutral	Highly Damaging
11	rs1299107729	NM_053056.2:c.46G>T, NP_444284.1:p.Ala16Ser	0.998	Probably Damaging	0.06	Tolerant	-1.74	Neutral	0.961	Disease	0.565	Disease	Highly Damaging
12	rs772857967	NM_053056.2:c.52C>G, NP_444284.1:p.Pro18Ala	0.001	Benign	0.75	Tolerant	-2.34	Neutral	0.695	Disease	0.293	Neutral	Benign
		NM_053056.2:c.52C>T, NP_444284.1:p.Pro18Ser	0.000	Benign	0.70	Tolerant	-2.26	Neutral	0.780	Disease	0.266	Neutral	Benign
13	rs1417631865	NM_053056.2:c.57T>A, NP_444284.1:p.Asp19Glu	0.987	Probably Damaging	0.05	Tolerant	-2.85	Deleterious	0.960	Disease	0.444	Neutral	Highly Damaging
14	rs766170770	NM_053056.2:c.61A>G,	0.645	Possibly	0.36	Tolerant	-2	Neutral	0.810	Disease	0.296	Neutral	Benign

		NP_444284.1:p.Asn21Asp		damaging									
15	rs753863475	NM_053056.2:c.67C>T, NP_444284.1:p.Leu23Phe	0.01	Benign	0.06	Tolerant	-2.11	Neutral	0.876	Disease	0.487	Neutral	Benign
16	rs1400812649	NM_053056.2:c.74A>G, NP_444284.1:p.Asp25Gly	0.876	Possibly damaging	0.01	Damaging	-4.51	Deleterious	0.986	Disease	0.800	Disease	Highly Damaging
17	rs2220247	NM_053056.2:c.88G>T, NP_444284.1:p.Ala30Ser,	0.000	Benign	0.47	Tolerant	0.79	Neutral	0.628	Disease Disease	0.187	Neutral	Benign
		NM_053056.2:c.88G>A, NP_444284.1:p.Ala30Thr	0.000	Benign	0.62	Tolerant	0.16	Neutral	0.658	Disease	0.208	Neutral	Benign
18	rs1415272481	NM_053056.2:c.89C>A, NP_444284.1:p.Ala30Asp	0.074	Benign	0.07	Tolerant	-0.43	Neutral	0.933	Disease	0.355	Neutral	Benign
19	rs746088878	NM_053056.2:c.106G>A, NP_444284.1:p.Glu36Lys	0.116	Benign	0.02	Damaging	-2.8	Deleterious	0.803	Disease	0.233	Neutral	Highly Damaging
20	rs1482952019	NM_053056.2:c.108G>T, NP_444284.1:p.Glu36Asp	0	Benign	0.30	Tolerant	-0.49	Neutral	0.897	Disease	0.546	Disease	Benign
21	rs1263446681	NM_053056.2:c.110C>G, NP_444284.1:p.Thr37Ser	0.001	Benign	0.02	Damaging	-0.73	Neutral	0.645	Disease	0.276	Neutral	Benign
22	rs780366497	NM_053056.2:c.114C>G, NP_444284.1:p.Cys38Trp	0.989	Probably Damaging	0.03	Damaging	-0.53	Neutral	0.959	Disease	0.600	Disease	Highly Damaging
23	rs749614691	NM_053056.2:c.122C>T, NP_444284.1:p.Ser41Leu	0.865	Possibly damaging	0.14	Tolerant	-3.23	Deleterious	0.825	Disease	0.574	Disease	Highly Damaging
24	rs1173840555	NM_053056.2:c.135C>A, NP_444284.1:p.Phe45Leu	0.09	Benign	0.06	Tolerant	-4.61	Deleterious	0.690	Disease	0.302	Neutral	Benign
25	rs747665638	NM_053056.2:c.136A>G, NP_444284.1:p.Lys46Glu	0.012	Benign	0.25	Tolerant	-1.27	Neutral	0.373	Neutral	NA	Unclassified	Benign
26	rs1332727287	NM_053056.2:c.148A>G, NP_444284.1:p.Lys50Glu	0	Benign	0.11	Tolerant	-1.38	Neutral	0.826	Disease	0.311	Neutral	Benign
27	rs772785280	NM_053056.2:c.153G>C, NP_444284.1:p.Glu51Asp	0	Benign	0.08	Tolerant	0.33	Neutral	0.410	Neutral	0.219	Neutral	
28	rs760341225	NM_053056.2:c.161C>G, NP_444284.1:p.Pro54Arg	1	Probably Damaging	0.00	Damaging	-6.6	Deleterious	0.928	Disease	0.700	Disease	Highly Damaging
29	rs765904377	NM_053056.2:c.166A>T, NP_444284.1:p.Met56Leu	0.14	Benign	0.09	Tolerant	-2.18	Neutral	0.956	Disease	0.535	Disease	Benign
30	rs748632355	NM_053056.2:c.185C>A, NP_444284.1:p.Thr62Asn,	0.06	Benign	0.45	Tolerant	-1.95	Neutral	0.904	Disease	0.388	Neutral	Benign

		NM_053056.2:c.185C>T, NP_444284.1:p.Thr62Ile	0.854	Possibly damaging	0.32	Tolerant	-2.96	Deleterious	0.955	Disease	0.705	Disease	Highly Damaging
31	rs1299820976	NM_053056.2:c.190A>C, NP_444284.1:p.Met64Leu	0.034	Benign	0.06	Tolerant	-2.23	Neutral	0.731	Disease	0.429	Neutral	Benign
32	rs759345822	NM_053056.2:c.197A>C, NP_444284.1:p.Glu66Ala,	0.981	Probably Damaging	0.00	Damaging	-4.67	Deleterious	0.917	Disease	0.588	Disease	Highly Damaging
		NM_053056.2:c.197A>G, NP_444284.1:p.Glu66Gly	0.994	Probably Damaging	0.00	Damaging	-5.3	Deleterious	0.943	Disease	0.737	Disease	Highly Damaging
33	rs769045064	NM_053056.2:c.206A>G, NP_444284.1:p.Glu69Gly	0.055	Benign	0.03	Damaging	-5.99	Deleterious	0.882	Disease	0.721	Disease	Highly Damaging
34	rs1381472659	NM_053056.2:c.220G>A, NP_444284.1:p.Glu74Lys	0.415	Benign	0.03	Damaging	-3.15	Deleterious	0.853	Disease	0.444	Neutral	Highly Damaging
35	rs1171564865	NM_053056.2:c.223G>A, NP_444284.1:p.Glu75Lys	0.224	Benign	0.01	Damaging	-3.09	Deleterious	0.932	Disease	0.528	Disease	Highly Damaging
36	rs778080996	NM_053056.2:c.226G>A, NP_444284.1:p.Glu76Lys	0.782	Possibly damaging	0.07	Tolerant	-3.25	Deleterious	0.979	Disease	0.495	Neutral	Highly Damaging
37	rs746530862	NM_053056.2:c.228G>C, NP_444284.1:p.Glu76Asp	0.001	Benign	0.55	Tolerant	-1.08	Neutral	0.842	Disease	0.399	Neutral	Benign
38	rs781047821	NM_053056.2:c.235C>T, NP_444284.1:p.Pro79Ser	0.851	Possibly damaging	0.01	Damaging	-6.25	Deleterious	0.864	Disease	0.483	Neutral	Highly Damaging
39	rs1188165799	NM_053056.2:c.241G>A, NP_444284.1:p.Ala81Thr	0.995	Possibly damaging	0.05	Tolerant	-2.79	Deleterious	0.913	Disease	0.885	Disease	Highly Damaging
40	rs1471534715	NM_053056.2:c.244A>G, NP_444284.1:p.Met82Val	0.002	Benign	0.41	Tolerant	-2.75	Deleterious	0.513	Disease	0.321	Neutral	Benign
41	rs1359042394	NM_053056.2:c.277C>A, NP_444284.1:p.Pro93Ser,	0.01	Benign	0.17	Tolerant	-5.11	Deleterious	0.514	Disease	0.233	Neutral	Benign
		NM_053056.2:c.277C>T, NP_444284.1:p.Pro93Thr	0.004	Benign	0.14	Tolerant	-5.37	Deleterious	0.577	Disease	0.489	Neutral	Benign
42	rs1419585038	NM_053056.2:c.278C>T, NP_444284.1:p.Pro93Leu	0.164	Benign	0.01	Damaging	-7.15	Deleterious	0.828	Disease	0.617	Disease	Highly Damaging
43	rs774091629	NM_053056.2:c.280G>T, NP_444284.1:p.Val94Leu	0	Benign	0.39	Tolerant	-0.84	Neutral	0.746	Disease	0.245	Neutral	Benign
44	rs760878237	NM_053056.2:c.285A>C, NM_053056.2:c.285A>G,	0.011	Benign	0.18	Tolerant	-2.52	Deleterious	0.729	Disease	0.342	Neutral	Benign

		NP_444284.1:p.Lys95Asn											
45	rs148113872	NM_053056.2:c.288G>C,<br>NM_053056.2:c.288G>T,<br>NP_444284.1:p.Lys96Asn	0.999	Probably Damaging	0.00	Damaging	-4.23	Deleterious	0.957	Disease	0.673	Disease	Highly Damaging
46	rs140967247	NM_053056.2:c.292C>A,<br>NP_444284.1:p.Arg98Ser	0.195	Benign	0.41	Tolerant	-2.13	Neutral	0.812	Disease	0.341	Neutral	Benign
47	rs1480433568	NM_053056.2:c.302T>G,<br>NP_444284.1:p.Leu101Arg	0.998	Probably Damaging	0.00	Damaging	-5.05	Deleterious	0.989	Disease	0.982	Disease	Highly Damaging
48	rs753115532	NM_053056.2:c.310G>T,<br>NP_444284.1:p.Ala104Ser,	0.987	Probably	0.22	Tolerant	-1.79	Neutral	0.902	Disease	0.498	Neutral	Benign
		NM_053056.2:c.310G>A,<br>NP_444284.1:p.Ala104Thr	0.999	Damaging	0.26	Tolerant	-2.46	Neutral	0.869	Disease	0.564	Disease	Highly Damaging
49	rs764727750	NM_053056.2:c.319A>C,<br>NP_444284.1:p.Met107Leu	0.003	Benign	0.49	Tolerant	-1.84	Neutral	0.391	Neutral	0.417	Neutral	Benign
50	rs751995000	NM_053056.2:c.324C>A,<br>NP_444284.1:p.Phe108Leu	0.104	Benign	0.48	Tolerant	-2.9	Neutral	0.774	Disease	0.266	Neutral	Benign
51	rs374998781	NM_053056.2:c.341A>G,<br>NP_444284.1:p.Lys114Arg	0.048	Benign	0.50	Tolerant	-1.21	Deleterious	0.746	Disease	0.240	Neutral	Benign
52	rs755854763	NM_053056.2:c.350T>A,<br>NP_444284.1:p.Ile117Asn	0.09	Benign	0.57	Tolerant	-2.25	Neutral	0.893	Disease	0.387	Neutral	Benign
53	rs1176401077	NM_053056.2:c.351C>G,<br>NP_444284.1:p.Ile117Met	0.059	Benign	0.14	Tolerant	-0.57	Neutral	0.893<br>0.883	Disease	0.387<br>0.622	Neutral<br>Disease	Benign
54	rs1469991905	NM_053056.2:c.361G>A,<br>NP_444284.1:p.Ala121Thr	0.909	Possibly damaging	0.10	Tolerant	-2.26	Neutral	0.915	Disease	0.601	Disease	Highly Damaging
55	rs768334579	NM_053056.2:c.364G>A,<br>NP_444284.1:p.Glu122Lys	0.787	Possibly damaging	0.02	Damaging	-2.95	Neutral	0.930	Disease	0.517	Disease	Highly Damaging
56	rs1373598994	NM_053056.2:c.365A>G,<br>NP_444284.1:p.Glu122Gly	0.289	Benign	0.01	Damaging	-5.42	Deleterious	0.808	Disease	0.530	Disease	Highly Damaging
57	rs1449765247	NM_053056.2:c.367A>C,<br>NP_444284.1:p.Lys123Gln	0.746	Possibly damaging	0.05	Damaging	-3.14	Deleterious	0.608	Disease	0.373	Neutral	Highly Damaging
58	rs774409065	NM_053056.2:c.368A>T,<br>NP_444284.1:p.Lys123Met	0.592	Possibly damaging	0.01	Damaging	-4.79	Deleterious	0.834	Disease	0.819	Disease	Highly Damaging
59	rs748059951	NM_053056.2:c.371T>C,<br>NP_444284.1:p.Leu124Pro	1	Probably Damaging	0.00	Damaging	-6.28	Deleterious	0.989	Disease	0.974	Disease	Highly Damaging
60	rs1329584649	NM_053056.2:c.376A>C,<br>NP_444284.1:p.Ile126Leu	0.003	Benign	0.14	Tolerant	-1.31	Deleterious	0.895	Disease	0.509	Disease	Highly Damaging
61	rs1375845153	NM_053056.2:c.379T>C,	0.999	Probably	0.01	Damaging	-4.35	Neutral	0.946	Disease	0.855	Disease	Highly

		NP_444284.1:p.Tyr127His		Damaging									Damaging
62	rs1312761797	NM_053056.2:c.382A>G, NP_444284.1:p.Thr128Ala	0.892	Possibly damaging	0.01	Damaging	-4.17	Deleterious	0.837	Disease	0.395	Neutral	Highly Damaging
63	rs771952150	NM_053056.2:c.385G>A, NP_444284.1:p.Asp129Asn	0.951	Possibly damaging	0.01	Damaging	-4.29	Deleterious	0.960	Disease	0.670	Disease	Highly Damaging
64	rs1050971	NM_053056.2:c.388A>G, NP_444284.1:p.Asn130Asp,	0.194	Benign	0.06	Tolerant	-3.65	Deleterious	0.923	Disease	0.916	Disease	Highly Damaging
		NM_053056.2:c.388A>T, NP_444284.1:p.Asn130Tyr	0.31	Benign	0.12	Tolerant	-4.91	Deleterious	0.648	Disease	0.257	Neutral	Benign
65	rs1131439	NM_053056.2:c.389A>G, NP_444284.1:p.Asn130Ser	0.196	Benign	0.10	Tolerant	-3.52	Deleterious	0.876	Disease	0.926	Disease	Highly Damaging
66	rs759551813	NM_053056.2:c.392C>T, NP_444284.1:p.Ser131Phe	0.982	Probably Damaging	0.00	Damaging	-4.95	Deleterious	0.956	Disease	0.691	Disease	Highly Damaging
67	rs866931401	NM_053056.2:c.398G>A, NP_444284.1:p.Arg133Gln,	0	Benign	0.30	Tolerant	-0.96	Neutral	0.794	Disease	0.382	Neutral	Benign
		NM_053056.2:c.398G>T, NP_444284.1:p.Arg133Leu	0	Benign	0.15	Tolerant	-2.19	Neutral	0.934	Disease	0.548	Disease	Benign
68	rs765498502	NM_053056.2:c.400C>T, NP_444284.1:p.Pro134Ser	0.185	Benign	0.57	Tolerant	-3.62	Deleterious	0.718	Disease	0.490	Neutral	Benign
69	rs763443319	NM_053056.2:c.403G>A, NP_444284.1:p.Glu135Lys	0.001	Benign	0.50	Tolerant	-1.2	Neutral	0.781	Disease	0.427	Neutral	Benign
70	rs764335132	NM_053056.2:c.404A>T, NP_444284.1:p.Glu135Val	0.003	Benign	0.10	Tolerant	-3.74	Deleterious	0.882	Disease	0.538	Disease	Highly Damaging
71	rs1184664065	NM_053056.2:c.407A>C, NP_444284.1:p.Glu136Ala	0.751	Possibly damaging	0.01	Tolerant	-4.63	Deleterious	0.896	Disease	0.490	Neutral	Benign
72	rs752157015	NM_053056.2:c.409C>A, NP_444284.1:p.Leu137Met	0.99	Probably Damaging	0.03	Damaging	-1.66	Neutral	0.935	Disease	0.823	Disease	Highly Damaging
73	rs1268871232	NM_053056.2:c.417A>C, NP_444284.1:p.Gln139His	0.001	Benign	0.13	Tolerant	-1.96	Neutral	0.806	Disease	0.669	Disease	Benign
74	rs1260695584	NM_053056.2:c.427C>T, NP_444284.1:p.Leu143Phe	0.322	Benign	0.05	Damaging	-3.07	Deleterious	0.832	Disease	0.824	Disease	Highly Damaging
75	rs766855822	NM_053056.2:c.428T>C, NP_444284.1:p.Leu143Pro	0.544	Possibly damaging	0.00	Damaging	-5.82	Deleterious	0.969	Disease	0.937	Disease	Highly Damaging
76	rs753296773	NM_053056.2:c.430C>G,	0.004	Benign	1.00	Tolerant	1.20	Neutral	0.254	Neutral	0.556	Disease	Benign

		NP_444284.1:p.Leu144Val												
77	rs374420164	NM_053056.2:c.442C>T, NP_444284.1:p.Leu148Phe	1	Probably Damaging	0.00	Damaging	-3.84	Deleterious	0.952	Disease	0.943	Disease	Highly Damaging
78	rs1203615889	NM_053056.2:c.454C>A, NP_444284.1:p.Leu152Met	0.992	Probably Damaging	0.08	Tolerant	-1.51	Neutral	0.641	Disease	0.284	Neutral	Benign
79	rs752345237	NM_053056.2:c.460G>C, NP_444284.1:p.Ala154Pro,	0.994	Probably Damaging	0.01	Damaging	-4.02	Deleterious	0.950	Disease	0.490	Neutral	Highly Damaging
		NM_053056.2:c.460G>T, NP_444284.1:p.Ala154Ser	0.087	Benign	0.43	Tolerant	-1.39	Neutral	0.679	Disease	0.235	Neutral	Benign
80	rs1292226646	NM_053056.2:c.463A>G, NP_444284.1:p.Met155Val	0.001	Benign	1.00	Tolerant	1.09	Neutral	0.460	Neutral	0.691	Disease	Benign
81	rs11263523	NM_053056.2:c.474C>A, NM_053056.2:c.474C>G, NP_444284.1:p.His158Gln	0.093	Benign	0.20	Tolerant	-4.34	Deleterious	0.950	Disease	0.685	Disease	Highly Damaging
82	rs781118003	NM_053056.2:c.475G>A, NP_444284.1:p.Asp159Asn	0.998	Probably Damaging	0.01	Damaging	-4.67	Deleterious	0.956	Disease	0.747	Disease	Highly Damaging
83	rs749340325	NM_053056.2:c.476A>G, NP_444284.1:p.Asp159Gly	0.993	Probably Damaging	0.00	Damaging	-6.59	Deleterious	0.915	Disease	0.648	Disease	Highly Damaging
84	rs1447014281	NM_053056.2:c.483T>G, NP_444284.1:p.Ile161Met	0.925	Possibly damaging	0.01	Damaging	-1.76	Neutral	0.937	Disease	0.699	Disease	Highly Damaging
85	rs1332977808	NM_053056.2:c.485A>G, NP_444284.1:p.Glu162Gly	0.304	Benign	0.06	Tolerant	-4.4	Deleterious	0.947	Disease	0.709	Disease	Highly Damaging
86	rs768767169	NM_053056.2:c.488A>G, NP_444284.1:p.His163Arg	0.927	Possibly damaging	0.02	Damaging	-5.56	Deleterious	0.904	Disease	0.811	Disease	Highly Damaging
87	rs537363548	NM_053056.2:c.490T>C, NP_444284.1:p.Phe164Leu	0.206	Benign	0.19	Tolerant	-0.87	Neutral	0.882	Disease	0.273	Neutral	Benign
88	rs1432185835	NM_053056.2:c.493C>G, NP_444284.1:p.Leu165Val	0.723	Possibly damaging	0.05	Tolerant	-2.54	Deleterious	0.882	Disease	0.511	Disease	Highly Damaging
89	rs1261290754	NM_053056.2:c.505C>T, NP_444284.1:p.Pro169Ser	0.167	Benign	0.21	Tolerant	-4.61	Deleterious	0.500	Disease	0.299	Neutral	Benign
90	rs548963461	NM_053056.2:c.509A>T, NP_444284.1:p.Glu170Val	0	Benign	0.58	Tolerant	2.49	Neutral	0.582	Disease	0.237	Neutral	Benign
91	rs747336419	NM_053056.2:c.510G>C, NP_444284.1:p.Glu170Asp	0	Benign	0.07	Tolerant	-0.86	Neutral	0.680	Disease	0.293	Neutral	Benign
92	rs1373641771	NM_053056.2:c.512C>T,	0.003	Benign	0.57	Tolerant	-1.21	Neutral	0.766	Disease	0.335	Neutral	Benign

		NP_444284.1:p.Ala171Val											
93	rs374062310	NM_053056.2:c.522C>G, NP_444284.1:p.Asn174Lys	0.01	Benign	1.00	Tolerant	-0.36	Neutral	0.586	Disease	NA	Unclassified	Benign
94	rs1175448240	NM_053056.2:c.523A>G, NP_444284.1:p.Lys175Glu	0.002	Benign	1.00	Tolerant	-1.43	Neutral	0.503	Disease	NA	Unclassified	Benign
95	rs777185874	NM_053056.2:c.526C>G, NP_444284.1:p.Gln176Glu	0	Benign	1.00	Tolerant	-0.42	Neutral	0.474	Neutral	NA	Unclassified	Benign
96	rs1433903280	NM_053056.2:c.527A>T, NP_444284.1:p.Gln176Leu	0	Benign	0.32	Tolerant	-2.41	Neutral	0.392	Neutral	NA	Unclassified	Benign
97	rs143479406	NM_053056.2:c.536G>A, NP_444284.1:p.Arg179His,	0.919	Possibly damaging	0.05	Tolerant	-3.84	Deleterious	0.907	Disease	NA	Unclassified	Highly Damaging
		NM_053056.2:c.536G>T, NP_444284.1:p.Arg179Leu	0.947	Possibly damaging	0.02	Damaging	-5.10	Deleterious	0.837	Disease	0.268	Neutral	Highly Damaging
98	rs1176469241	NM_053056.2:c.543C>A, NP_444284.1:p.His181Gln	0.903	Possibly damaging	0.03	Damaging	-7.39	Deleterious	0.898	Disease	0.426	Neutral	Highly Damaging
99	rs1434771092	NM_053056.2:c.547C>A, NP_444284.1:p.Gln183Lys	0.237	Benign	0.04	Damaging	-3.08	Deleterious	0.881	Disease	0.508	Disease	Highly Damaging
100	rs1173908293	NM_053056.2:c.551C>G, NP_444284.1:p.Thr184Ser	0.103	Benign	0.08	Tolerant	-3.53	Deleterious	0.766	Disease	0.423	Neutral	Benign
101	rs763697017	NM_053056.2:c.556G>A, NP_444284.1:p.Val186Ile	0.002	Benign	1.00	Tolerant	0.29	Neutral	0.346	Neutral	0.284	Neutral	Benign
102	rs1252289930	NM_053056.2:c.560C>T, NP_444284.1:p.Ala187Val	0.912	Possibly damaging	0.06	Tolerant	-3.58	Deleterious	0.940	Disease	0.440	Neutral	Highly Damaging
103	rs751315372	NM_053056.2:c.562C>T, NP_444284.1:p.Leu188Phe	1	Probably Damaging	0.00	Damaging	-3.76	Deleterious	0.973	Disease	0.838	Disease	Highly Damaging
104	rs757229078	NM_053056.2:c.566G>A, NP_444284.1:p.Cys189Tyr	1	Probably Damaging	0.00	Damaging	-9.72	Deleterious	0.982	Disease	0.706	Disease	Highly Damaging
105	rs534553548	NM_053056.2:c.568G>T, NP_444284.1:p.Ala190Ser	0.894	Possibly damaging	0.12	Tolerant	-2.2	Neutral	0.942	Disease	0.635	Disease	Highly Damaging
106	rs201012923	NM_053056.2:c.577G>A, NP_444284.1:p.Val193Met	0.013	Benign	0.14	Tolerant	-0.55	Neutral	0.871	Disease	0.415	Neutral	Benign
107	rs1282637304	NM_053056.2:c.582G>T, NP_444284.1:p.Lys194Asn	0.02	Benign	0.34	Tolerant	-2.21	Neutral	0.841	Disease	0.485	Neutral	Benign
108	rs759570740	NM_053056.2:c.593A>G, NP_444284.1:p.Asn198Ser	0.02	Benign	0.60	Tolerant	-1.22	Neutral	0.769	Disease	0.386	Neutral	Benign
109	rs1222442441	NM_053056.2:c.596C>T, NP_444284.1:p.Pro199Leu	0.998	Probably Damaging	0.00	Damaging	-7.42	Deleterious	0.776	Disease	0.377	Neutral	Highly Damaging

110	rs1353308163	NM_053056.2:c.598C>T, NP_444284.1:p.Pro200Ser	1	Probably Damaging	0.00	Damaging	-7.2	Deleterious	0.969	Disease	0.831	Disease	Highly Damaging
111	rs893823618	NM_053056.2:c.607G>T, NP_444284.1:p.Val203Leu	0.004	Benign	0.07	Tolerant	-1.29	Neutral	0.853	Disease	0.357	Neutral	Benign
112	rs751664590	NM_053056.2:c.610G>A, NP_444284.1:p.Ala204Thr	1	Probably Damaging	0.00	Damaging	-3.6	Deleterious	0.974	Disease	0.895	Disease	Highly Damaging
113	rs1193747086	NM_053056.2:c.613G>T, NP_444284.1:p.Ala205Ser	0.996	Probably Damaging	0.09	Tolerant	-1.29	Neutral	0.959	Disease	0.756	Disease	Highly Damaging
114	rs1271260640	NM_053056.2:c.614C>T, NP_444284.1:p.Ala205Val	1	Probably Damaging	0.01	Damaging	-2.42	Neutral	0.957	Disease	0.689	Disease	Highly Damaging
115	rs1011426441	NM_053056.2:c.622G>A, NP_444284.1:p.Val208Met	0.977	Probably Damaging	0.09	Tolerant	-1.82	Neutral	0.781	Disease	0.379	Neutral	Benign
116	rs750639632	NM_053056.2:c.626T>C, NP_444284.1:p.Val209Ala	0	Benign	1.00	Tolerant	0.72	Neutral	0.697	Disease	0.325	Neutral	Benign
117	rs756509430	NM_053056.2:c.631G>A, NP_444284.1:p.Ala211Thr	1	Probably Damaging	0.00	Damaging	-3.47	Deleterious	0.968	Disease	0.706	Disease	Highly Damaging
118	rs112525097	NM_053056.2:c.648C>G, NP_444284.1:p.Asn216Lys	0	Benign	0.63	Tolerant	-1.27	Neutral	0.863	Disease	0.376	Neutral	Benign
119	rs149457002	NM_053056.2:c.656G>A, NP_444284.1:p.Ser219Asn	0	Benign	0.52	Tolerant	-1.22	Neutral	0.876	Disease	0.289	Neutral	Benign
120	rs1404273153	NM_053056.2:c.658C>T, NP_444284.1:p.Pro220Ser	0	Benign	0.89	Tolerant	0.44	Neutral	0.805	Disease	NA	Unclassified	Benign
121	rs747703578	NM_053056.2:c.659C>T, NP_444284.1:p.Pro220Leu	0	Benign	0.35	Tolerant	-1.96	Neutral	0.818	Disease	NA	Unclassified	Benign
122	rs1412188214	NM_053056.2:c.661A>C, NP_444284.1:p.Asn221His	0.159	Benign	0.12	Tolerant	-2.37	Neutral	0.920	Disease	0.652	Disease	Benign
123	rs746640562	NM_053056.2:c.670C>A, NP_444284.1:p.Leu224Met	0.034	Benign	0.22	Tolerant	-0.36	Neutral	0.918	Disease	0.649	Disease	Benign
124	rs776600964	NM_053056.2:c.677A>G, NP_444284.1:p.Tyr226Cys,	0	Benign	0.18	Tolerant	-1.42	Neutral	0.853	Disease	0.235	Neutral	Benign
		NM_053056.2:c.677A>T, NP_444284.1:p.Tyr226Phe	0.002	Benign	0.70	Tolerant	-0.93	Neutral	0.816	Disease	0.262	Neutral	Benign
125	rs1222446202	NM_053056.2:c.682C>T, NP_444284.1:p.Arg228Cys	0.004	Benign	0.05	Tolerant	-3.18	Deleterious	0.754	Disease	0.203	Neutral	Benign
126	rs200179137	NM_053056.2:c.683G>A, NP_444284.1:p.Arg228His	0	Benign	0.13	Tolerant	-1.33	Neutral	0.909	Disease	0.572	Disease	Benign

127	rs984643266	NM_053056.2:c.689C>T, NP_444284.1:p.Thr230Ile,	0.476	Possibly damaging	0.60	Tolerant	-2.52	Deleterious	0.940	Disease	0.570	Disease	Highly Damaging
		NM_053056.2:c.689C>A, NP_444284.1:p.Thr230Lys	0.998	Probably Damaging	0.06	Tolerant	-3.71	Deleterious	0.934	Disease	0.255	Neutral	Highly Damaging
128	rs745779714	NM_053056.2:c.691C>T, NP_444284.1:p.Arg231Cys,	0.495	Possibly damaging	0.03	Damaging	-3.35	Deleterious	0.889	Disease	0.410	Neutral	Highly Damaging
		NM_053056.2:c.691C>G, NP_444284.1:p.Arg231Gly,	0	Benign	0.20	Tolerant	-1.83	Neutral	0.860	Disease	0.325	Neutral	Benign
		NM_053056.2:c.691C>A, NP_444284.1:p.Arg231Ser	0	Benign	0.40	Tolerant	-0.58	Neutral	0.838	Disease	0.403	Neutral	Benign
129	rs1228811654	NM_053056.2:c.697C>A, NP_444284.1:p.Leu233Ile	0.998	Probably Damaging	0.05	Tolerant	-1.63	Neutral	0.886	Disease	0.507	Disease	Highly Damaging
130	rs1165177408	NM_053056.2:c.700T>A, NP_444284.1:p.Ser234Thr	0.001	Benign	0.89	Tolerant	-1.08	Neutral	0.716	Disease	0.479	Neutral	Benign
131	rs1423874357	NM_053056.2:c.712A>C, NP_444284.1:p.Lys238Gln	0	Benign	0.23	Tolerant	-1.09	Neutral	0.723	Disease	0.506	Disease	Benign
132	rs760907398	NM_053056.2:c.714G>A, NM_053056.2:c.714G>C, NP_444284.1:p.Lys238Asn	0	Benign	0.89	Tolerant	0.2	Neutral	0.777	Disease	0.547	Disease	Benign
133	rs777225097	NM_053056.2:c.722C>T, NP_444284.1:p.Pro241Leu	0.001	Benign	0.33	Tolerant	-2.98	Deleterious	0.507	Disease	0.265	Neutral	Benign
134	rs913470506	NM_053056.2:c.733C>T, NP_444284.1:p.Arg245Trp	1	Probably Damaging	0.00	Damaging	-5.99	Deleterious	0.933	Disease	0.875	Disease	Highly Damaging
135	rs925960764	NM_053056.2:c.736G>T, NP_444284.1:p.Ala246Ser	0.008	Benign	0.23	Tolerant	-1.31	Neutral	0.797	Disease	0.373	Neutral	Benign
136	rs1417190579	NM_053056.2:c.740G>T, NP_444284.1:p.Cys247Phe,	1	Probably Damaging	0.00	Damaging	-9.74	Deleterious	0.956	Disease	0.310	Neutral	Highly Damaging
		NM_053056.2:c.740G>A, NP_444284.1:p.Cys247Tyr	1	Probably Damaging	0.00	Damaging	-9.63	Deleterious	0.980	Disease	0.427	Neutral	Highly Damaging
137	rs1446903472	NM_053056.2:c.748C>A, NP_444284.1:p.Gln250Lys	0.92	Possibly damaging	0.02	Damaging	-3.28	Deleterious	0.878	Disease	0.246	Neutral	Highly Damaging

138	rs1377678585	NM_053056.2:c.749A>G, NP_444284.1:p.Gln250Arg	0.516	Possibly damaging	0.11	Tolerant	-3.24	Deleterious	0.782	Disease	0.245	Neutral	Highly Damaging
139	rs1173812710	NM_053056.2:c.754G>A, NP_444284.1:p.Glu252Lys	1	Probably Damaging	0.00	Damaging	-3.58	Deleterious	0.946	Disease	0.313	Neutral	Highly Damaging
140	rs750012493	NM_053056.2:c.773G>C, NP_444284.1:p.Ser258Thr	0.23	Benign	0.01	Damaging	-2.06	Deleterious	0.752	Disease	0.180	Neutral	Highly Damaging
141	rs755986542	NM_053056.2:c.778C>T, NP_444284.1:p.Arg260Cys	0.262	Benign	0.11	Tolerant	-3.86	Neutral	0.942	Disease	0.706	Disease	Benign
142	rs779733976	NM_053056.2:c.779G>A, NP_444284.1:p.Arg260His	0.99	Probably Damaging	0.13	Tolerant	-2.41	Deleterious	0.907	Disease	0.457	Neutral	Highly Damaging
143	rs749069429	NM_053056.2:c.782A>G, NP_444284.1:p.Gln261Arg,	0.007	Benign	0.07	Tolerant	-2.06	Neutral	0.765	Disease	0.159	Neutral	Benign
		NM_053056.2:c.782A>C, NP_444284.1:p.Gln261Pro	0.004	Benign	0.08	Tolerant	-3.36	Deleterious	0.950	Disease	0.453	Neutral	Benign
145	rs1306960346	NM_053056.2:c.787C>G, NP_444284.1:p.Gln263Glu	0.008	Benign	0.23	Tolerant	-1.29	Neutral	0.816	Disease	0.212	Neutral	Benign
146	rs768582800	NM_053056.2:c.790C>A, NP_444284.1:p.Gln264Lys	0.035	Benign	0.36	Tolerant	-1.89	Neutral	0.841	Disease	0.166	Neutral	Benign
147	rs1225491115	NM_053056.2:c.797T>C, NP_444284.1:p.Met266Thr	0	Benign	0.60	Tolerant	-0.01	Neutral	0.571	Disease	0.184	Neutral	Benign
148	rs377271027	NM_053056.2:c.800A>G, NP_444284.1:p.Asp267Gly	0	Benign	0.40	Tolerant	-1.25	Neutral	0.636	Disease	0.288	Neutral	Benign
149	rs1323012735	NM_053056.2:c.802C>A, NP_444284.1:p.Pro268Thr	0.067	Benign	0.58	Tolerant	-1.43	Neutral	0.595	Disease	0.219	Neutral	Benign
150	rs1252799046	NM_053056.2:c.808G>A, NP_444284.1:p.Ala270Thr	0.033	Benign	0.61	Tolerant	-0.66	Neutral	0.655	Disease	0.223	Neutral	Benign
151	rs770991095	NM_053056.2:c.809C>T, NP_444284.1:p.Ala270Val	0.033	Benign	0.33	Tolerant	-0.75	Neutral	0.357	Neutral	0.246	Neutral	Benign
152	rs759765773	NM_053056.2:c.811G>A, NP_444284.1:p.Ala271Thr	0	Benign	0.77	Tolerant	0.72	Neutral	0.402	Neutral	0.150	Neutral	Benign
153	rs770021448	NM_053056.2:c.812C>A, NP_444284.1:p.Ala271Asp	0	Benign	0.68	Tolerant	-0.58	Neutral	0.681	Disease	0.125	Neutral	Benign
154	rs775723921	NM_053056.2:c.814G>A, NP_444284.1:p.Glu272Lys	0.007	Benign	0.83	Tolerant	0.24	Neutral	0.590	Disease	0.144	Neutral	Benign
155	rs764757265	NM_053056.2:c.821A>T, NP_444284.1:p.Glu274Val	0.001	Benign	0.28	Tolerant	-0.98	Neutral	0.649	Disease	0.378	Neutral	Benign

156	rs752108957	NM_053056.2:c.823G>C, NP_444284.1:p.Glu275Gln	0.005	Benign	0.55	Tolerant	-0.2	Neutral	0.398	Neutral	0.140	Neutral	Benign
157	rs1436128793	NM_053056.2:c.827A>T, NP_444284.1:p.Glu276Val	0.047	Benign	0.15	Tolerant	-1.14	Neutral	0.711	Disease	0.484	Neutral	Benign
158	rs200912411	NM_053056.2:c.836A>C, NP_444284.1:p.Glu279Ala	0	Benign	0.60	Tolerant	-1.06	Neutral	0.383	Neutral	0.188	Neutral	Benign
159	rs749899296	NM_053056.2:c.845A>G, NP_444284.1:p.Asp282Gly,	0.002	Benign	0.11	Tolerant	-1.29	Neutral	0.691	Disease	0.219	Neutral	Benign
		NM_053056.2:c.845A>T, NP_444284.1:p.Asp282Val	0.425	Benign	0.03	Damaging	-2.72	Deleterious	0.648	Disease	0.209	Neutral	Highly Damaging
160	rs755753468	NM_053056.2:c.847C>G, NP_444284.1:p.Leu283Val	0.037	Benign	0.45	Tolerant	-0.80	Neutral	0.387	Neutral	0.146	Neutral	Benign
161	rs754752470	NM_053056.2:c.853T>A, NP_444284.1:p.Cys285Ser	0.001	Benign	1	Tolerant	-0.72	Neutral	0.496	Neutral	0.272	Neutral	Benign
162	rs1168483993	NM_053056.2:c.854G>T, NP_444284.1:p.Cys285Phe	0.28	Benign	0.56	Tolerant	-3.4	Deleterious	0.923	Disease	0.496	Neutral	Benign
163	rs771951669	NM_053056.2:c.865G>A, NP_444284.1:p.Asp289Asn	1	Probably Damaging	0.01	Damaging	-4.27	Deleterious	0.834	Disease	0.184	Neutral	Highly Damaging
164	rs781165229	NM_053056.2:c.867C>A, NM_053056.2:c.867C>T, NP_444284.1:p.Asp289Glu	1	Probably Damaging	0.03	Damaging	-3.35	Deleterious	0.842	Disease	0.203	Neutral	Highly Damaging
165	rs769921935	NM_053056.2:c.868G>C, NP_444284.1:p.Val290Leu,	0.99	Probably Damaging	0.09	Tolerant	-2.19	Neutral	0.821	Disease	0.317	Neutral	Benign
		NM_053056.2:c.868G>A, NP_444284.1:p.Val290Met	0.998	Probably Damaging	0.00	Damaging	-2.29	Neutral	0.788	Disease	0.475	Neutral	Highly Damaging
166	rs1292246537	NM_053056.2:c.871C>T, NP_444284.1:p.Arg291Trp	0.999	Probably Damaging	0.03	Damaging	-4.86	Deleterious	0.875	Disease	0.799	Disease	Highly Damaging
167	rs775768459	NM_053056.2:c.872G>A, NP_444284.1:p.Arg291Gln,	0.325	Benign	0.31	Tolerant	-1.3	Neutral	0.570	Disease	0.368	Neutral	Benign
		NM_053056.2:c.872G>T, NP_444284.1:p.Arg291Leu	0.212	Benign	0.81	Tolerant	-3.88	Deleterious	0.863	Disease	NA	Unclassified	Benign
168	rs535957987	NM_053056.2:c.876C>G, NP_444284.1:p.Asp292Glu	0.994	Probably Damaging	0.15	Tolerant	-2.56	Deleterious	0.785	Disease	0.257	Neutral	Highly damaging

169	rs1225090625	NM_053056.2:c.877G>A, NP_444284.1:p.Val293Met	0.999	Probably Damaging	0.08	Tolerant	-2.07	Neutral	0.709	Disease	0.342	Neutral	Benign

[a] PolyPhen-2= Polymorphism Phenotyping v2, Scores near to 1 are more confidently predicting the SNP to be damaging. http://genetics.bwh.harvard.edu/pph2/

[b] SIFT= Sorting Intolerant From Tolerant, SNP scores near 0.00 are more predicted as damaging. http://sift.bii.a-star.edu.sg/

[c] PROVEAN= Protein Variation Effect Analyzer, cut off -2.5, scores equal to or above this threshold are predicting the SNP as deleterious http://provean.jcvi.org/index.php

[d] SNP & GO= scores equal or above 0.5 are predicting the SNP as diseased http://snps.biofold.org/snps-and-go/snps-and-go.html

[e] PANTHER= PANTHER scores are given along with SNP & GO scores, scores equal or above 0.5 are predicting the SNP as diseased http://snps.biofold.org/snps-and-go/snps-and-go.html

[f] SNPs predicted as damaging by three or more tools are classified as "highly damaging" and selected for further *in silico* analysis (colored as red).

Supplementary Table 3. SNPeffect web server molecular phenotypic prediction

**SNP rs ID and amino acid change**	TANGO		WALTZ		LIMBO	
	Scores	Prediction	Scores	Prediction	Scores	Prediction
**rs557545630** Arg15Ser	-2.17	does not affect the aggregation tendency of cyclin D1	0.08	does not affect the amyloid propensity of cyclin D1	0.00	does not affect the chaperone binding tendency of cyclin D1
**rs534553548** Ala190Ser	-141.12	decreases the aggregation tendency of cyclin D1	8.07	does not affect the amyloid propensity of cyclin D1	0.00	does not affect the chaperone binding tendency of cyclin D1
**rs535957987** Asp292Glu	0.00	does not affect the aggregation tendency of cyclin D1	0.00	does not affect the amyloid propensity of cyclin D1	0.00	does not affect the chaperone binding tendency of cyclin D1
**rs143479406** Arg179His,	-2.01	does not affect the aggregation tendency of cyclin D1	0.10	does not affect the amyloid propensity of cyclin D1	0.00	does not affect the chaperone binding tendency of cyclin D1
**rs143479406** Arg179Leu	-2.19	does not affect the aggregation tendency of cyclin D1	5.85	does not affect the amyloid propensity of cyclin D1	-0.20	does not affect the chaperone binding tendency of cyclin D1
